# Orthogonal glycolytic pathway enables directed evolution of noncanonical cofactor oxidase

**DOI:** 10.1038/s41467-022-35021-x

**Published:** 2022-11-26

**Authors:** Edward King, Sarah Maxel, Yulai Zhang, Karissa C. Kenney, Youtian Cui, Emma Luu, Justin B. Siegel, Gregory A. Weiss, Ray Luo, Han Li

**Affiliations:** 1 Department of Molecular Biology and Biochemistry, University of California Irvine, Irvine, CA 92697 USA; 2 Department Chemical and Biomolecular Engineering University of California Irvine, Irvine, CA 92697 USA; 3 Department of Chemistry, University of California Irvine, Irvine, CA 92697 USA; 4 Genome Center, University of California Davis, Davis, CA 95616 USA; 5 Department of Chemistry, Molecular Medicine University of California, Davis, Davis, CA USA; 6 Department of Biochemistry and Molecular Medicine University of California, Davis, Davis, CA USA; 7 Department of Pharmaceutical Sciences, University of California Irvine, Irvine, CA 92697 USA; 8 Department Materials Science and Engineering, University of California Irvine, Irvine, CA 92697 USA; 9 Department of Biomedical Engineering, University of California Irvine, Irvine, CA 92697 USA

**Keywords:** Protein design, Synthetic biology, Microbiology techniques, Applied microbiology

## Abstract

Noncanonical cofactor biomimetics (NCBs) such as nicotinamide mononucleotide (NMN^+^) provide enhanced scalability for biomanufacturing. However, engineering enzymes to accept NCBs is difficult. Here, we establish a growth selection platform to evolve enzymes to utilize NMN^+^-based reducing power. This is based on an orthogonal, NMN^+^-dependent glycolytic pathway in *Escherichia coli* which can be coupled to any reciprocal enzyme to recycle the ensuing reduced NMN^+^. With a throughput of >10^6^ variants per iteration, the growth selection discovers a *Lactobacillus pentosus* NADH oxidase variant with ~10-fold increase in NMNH catalytic efficiency and enhanced activity for other NCBs. Molecular modeling and experimental validation suggest that instead of directly contacting NCBs, the mutations optimize the enzyme’s global conformational dynamics to resemble the WT with the native cofactor bound. Restoring the enzyme’s access to catalytically competent conformation states via deep navigation of protein sequence space with high-throughput evolution provides a universal route to engineer NCB-dependent enzymes.

## Abstract (web summary)

Engineering enzymes to accept noncanonical cofactor biomimetics is difficult. Here, the authors establish a self-sufficient growth selection method and demonstrate its application in engineering the *Lactobacillus pentosus* NADH oxidase to efficiently recycle reduced nicotinamide mononucleotide (NMNH).

## Introduction

Noncanonical cofactor biomimetics (NCBs) possess the reactive moieties essential for catalysis and carry several structural modifications that distinguish them from the natural cofactors nicotinamide adenine dinucleotide NAD(H) and nicotinamide adenine dinucleotide phosphate NADP(H)^1–3^. These NCBs offer distinct advantages over NAD(P)/H such as lower production cost^4^, expanded chemistries^5,6^, and, in some cases, improved stability^7^; NCBs also have the potential to minimize crosstalk between engineered pathways and native metabolism^8–10^(Supplementary Table 1). Some NCBs are metabolites that naturally exist in the cells^8^ or consist of natural building blocks^11^, which suggests their potential of being renewably biosynthesized. In particular, the biosynthesizable noncanonical cofactor nicotinamide mononucleotide NMN(H) mimics the conventional, native cofactors, but with a truncated structure lacking the adenosine binding handle typically involved with enzyme recognition. In *E. coli* whole cells and crude cell lysates, NMN^+^ can enable specific electron delivery^8^ and eliminate the need for extensive strain engineering as demonstrated by the respective 86% and 97–100% purity achieved through the NMNH-dependent whole cell and crude lysate production of the aldehyde citronellal^12^. In biotransformation in vitro, NMN^+^ can support a diverse range of industrially important chemistries with high productivity and long-term stability^8^.

To realize the potential of NCBs in wide-spread applications, tools must be developed to rapidly obtain efficient and diverse enzymes that can utilize NCBs^1,5,6,8,13,14^. Colorimetric assays in 96-well plates have been the predominant method for screening altered cofactor preferences^15^. Limited throughput with this method has motivated the development of efficient agar-based screening platforms^8,9,16^, which have been implemented to screen for NMN^+^ activity through 6-rounds of directed evolution to successfully identify a dehydrogenase with a 50-fold improvement of catalytic efficiency with NMN^+^^16^. While this method by Huang et al.^16^ greatly enhanced the throughput of screening for NCB activity (10^5^ per round), the required 70 °C heat treatment excludes application for mesophilic enzymes. Recent expansions of redox-based growth selections highlight the practical use of these platforms for engineering diverse cofactor-dependent enzymes with cell growth as an easy readout of activity^17–25^. While these selections can increase throughput to >10^6^, a link between the desired enzyme activity and a life-essential function is needed.

We previously demonstrated the initial proof-of-concept that cell growth can be coupled to NMN^+^ regeneration by wiring an engineered NMN^+^-specific glucose dehydrogenase (GDH Ortho) into *E. coli* central metabolism^8^. In this current work, the growth phenotype has been leveraged to develop a high-throughput selection platform with tunable sensitivity, a wider growth dynamic range, and the option to entirely omit exogenous NMN^+^ supplementation to select for enzymes that function with the physiologically relevant levels of NMN^+^ produced in vivo.

As a proof-of-concept, we engineered the *Lactobacillus pentosus* NADH oxidase (*Lp* Nox) to efficiently recycle NMNH using this selection platform. Water-forming NADH oxidases (Nox) are commonly utilized in regenerating the oxidized redox cofactors^2,3,14,26^. The wild-type *Lp* Nox was previously demonstrated to oxidize N-methyl-1,4-dihydronicotinamide (MNAH) and N-benzyl-1,4-dihydronicotinamide (BNAH), though at low catalytic efficiencies^26^. Using the growth-based selection platform developed in this work, we identified mutations from site-saturated mutagenesis and random mutagenesis libraries that improve *Lp* Nox’s capability and stability in recycling NMNH. The best variant, LP 3-EP features a ~10-fold increased catalytic activity toward NMNH, and interestingly also exhibits a ~2-4-fold increased activity toward MNAH and BNAH. To understand the mechanism for the improved activity toward NCBs, we perform experimental characterization and computational modeling. Our data suggest that wild-type *Lp* Nox suffers from excessive conformational flexibility when using NCBs, preventing productive catalysis. The mutations that we discover through growth selections, which do not directly interact with the cofactors, promote dense packing in the region where the adenosine of the native cofactor would bind, and restore conformational flexibility to favor sampling catalytically relevant states.

Here, we show emergent design principles that may guide future engineering for activity with NCBs. The growth selection platform we report in this study will enable the implementation of these principles in diverse enzymes, which would be otherwise unattainable without an evolution-based and target enzyme-agnostic method.

## Results

### Development and validation of NMNH-dependent growth selection

**Fig. 1:**
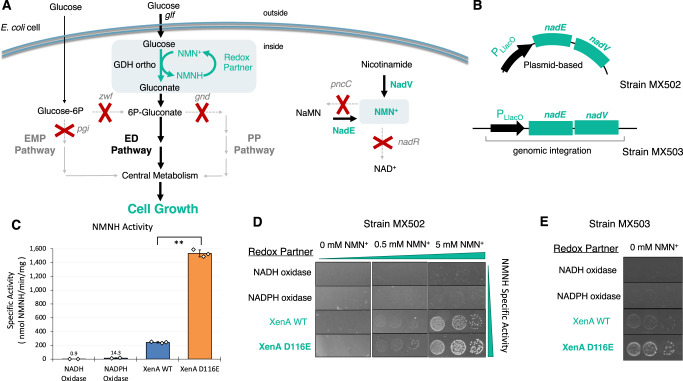
NMN^+^ regeneration drives dynamic glucose-dependent cell growth. **A** Engineered *E. coli* ED pathway couples cycling of NMN(H) to alternative glucose metabolism required for growth. Modified NMN^+^ pathways enhance intracellular NMN^+^ accumulation. Expression (green) of NAD^+^ synthase (*Ft* NadE) and nicotinate phosphoribosyltransferase (*Ft* NadV) with nicotinamide supplementation increases NMN^+^ availability, while deletion (grey) of *pncC* and *nadR* minimizes NMN^+^ degradation. **B** Reconfiguration of expression systems between strain MX502 and MX503. Strain MX502 requires three plasmids to support growth. In strain MX503, the *Ft* NadEV operon was integrated into the genome. **C** Specific activities of redox partners toward NMNH. From left to right, redox partners were NADH Oxidase (*Lb* Nox), NADPH Oxidase (TP Nox), XenA WT and XenA D116E (Both broad specificity). For XenA WT (blue) and XenA D116E (orange), *n* = 3 biologically independent replicates. For all other samples, *n* = 2 biologically independent replicates. For statistics, XenA WT to XenA D116E, *p* = 0.00054. **D** Validation of selection pressure in MX502. Four different redox partners were expressed in MX502 strains with 20 g/L glucose as the sole carbon source. Growth of strains was dependent on NMNH activity of assayed redox partners and the concentration of NMN^+^ supplemented in the media. **E** Validation of selection pressure in MX503. Reconfigured selection strain MX503 is capable of glucose-dependent growth without exogenous NMN^+^ addition. The limited NMN^+^ availability provided a more stringent selection condition compared to MX502 (Fig. 1D) and amplified growth differences between the XenA variants. Data are presented as mean ± standard deviation. The statistical significance was determined by two-tail *t*-tests (**: *p* < 0.005). Source data is provided as a Source Data file.

The selection platform utilized in this work is based on an *E. coli* strain that was engineered with disrupted natural glucose metabolism. The deletion of phosphoglucose isomerase *pgi* and glucose-6-phosphate 1-dehydrogenase *zwf* genes eliminates conventional glycolytic routes in the Embden-Meyerhof-Parnas (EMP) and pentose phosphate (PP) pathways, respectively. The resulting strain is unable to grow in minimal glucose media without the installation of an alternate channel to the life-essential central metabolism (Fig. 1A).

In this system, conversion of glucose to gluconate is controlled by the availability of NMN^+^ via an orthogonal NMN^+^-dependent glucose dehydrogenase (GDH Ortho). As NMN^+^ is reduced to NMNH by GDH Ortho, its supply becomes deficient and a redox cycling partner to oxidize NMNH is required to regenerate the supply, restoring carbon flux through the Entner Doudoroff (ED) pathway. While the initial iteration of this strain^8^ demonstrated a clear dependence of growth on the presence of NMN^+^, GDH Ortho, and an NMNH-dependent cycling partner, its practicality as a selection platform was limited. To improve utility, we reconfigured the strain to (1) expand the dynamic range of growth, ensuring high and low-activity redox partners are distinguishable, and (2) expressed the redox partner gene from a standalone plasmid, to allow interchangeability of different engineering targets. The modified *E. coli* selection strain, MX502 (Supplementary Table 2), carries two plasmids (pLM106 + pLS502), which possess the genes for initial glucose entry (GDH Ortho and a glucose facilitated diffusion porin from *Zymomonas mobilis, Zm Glf*), and NMN^+^ intracellular synthesis (*Francisella tularensis* NAD^+^ synthase, *Ft* NadE, and *F. tularensis* Nicotinate phosphoribosyltransferase, *Ft* NadV) (Fig. 1A, B)^8^. These two supporting plasmids have low to medium copy numbers to minimize growth burden. A third, high-copy plasmid is used to introduce various redox partners.

To benchmark the optimized strain’s sensitivity to NMNH-dependent activity, we tested four individual redox partners: an NADH-specific oxidase from *Lactobacillus brevis* (*Lb* Nox) (Supplementary Fig. 1A), an NADPH-specific oxidase^27^ (triphosphopyridine nucleotide oxidase, TP Nox) (Supplementary Fig. 1B) an enoate reductase (XenA) from *Pseudomonas putida* with broad cofactor specificity^13^ including NMNH (Supplementary Fig. 1B), and a mutant XenA D116E that demonstrates enhanced activity towards NMNH compared to the wild-type XenA (Fig. 1C, Supplementary Fig. 1C). After transformation into MX502, cell growth was characterized on solid selection media with varying NMN^+^ supplementation (Fig. 1D). Growth of cells expressing the NADH and NADPH-dependent oxidase was negligible, which highlighted the selection’s high specificity in presenting only the NMNH-utilizing redox partner, but not those cycling partners specific to the native redox cofactors in the cells. Additionally, expression of NMNH-active XenA facilitated growth in a dose-dependent manner with NMN^+^ supplementation, and XenA variant D116E sustained stronger growth (Fig. 1D, Supplementary Fig. 2). This growth trend aligned well with the redox partners’ NMNH-oxidizing activity and validated that the readout of growth can distinguish candidate enzymes with increased NMNH activity.

### Development of a self-sufficient NMNH-dependent growth selection

NMN^+^ can be synthesized intracellularly from inexpensive feedstocks using engineered metabolic pathways^28–31^ (Supplementary Table 1), which eliminates the cost of cofactor supplementation and makes the whole-cell biotransformation more scalable. The challenge is to rapidly obtain NMN(H)-dependent enzymes with suitable K_m_ values that directly function in the self-sufficient, NMN^+^-synthesizing strains.

**Fig. 2:**
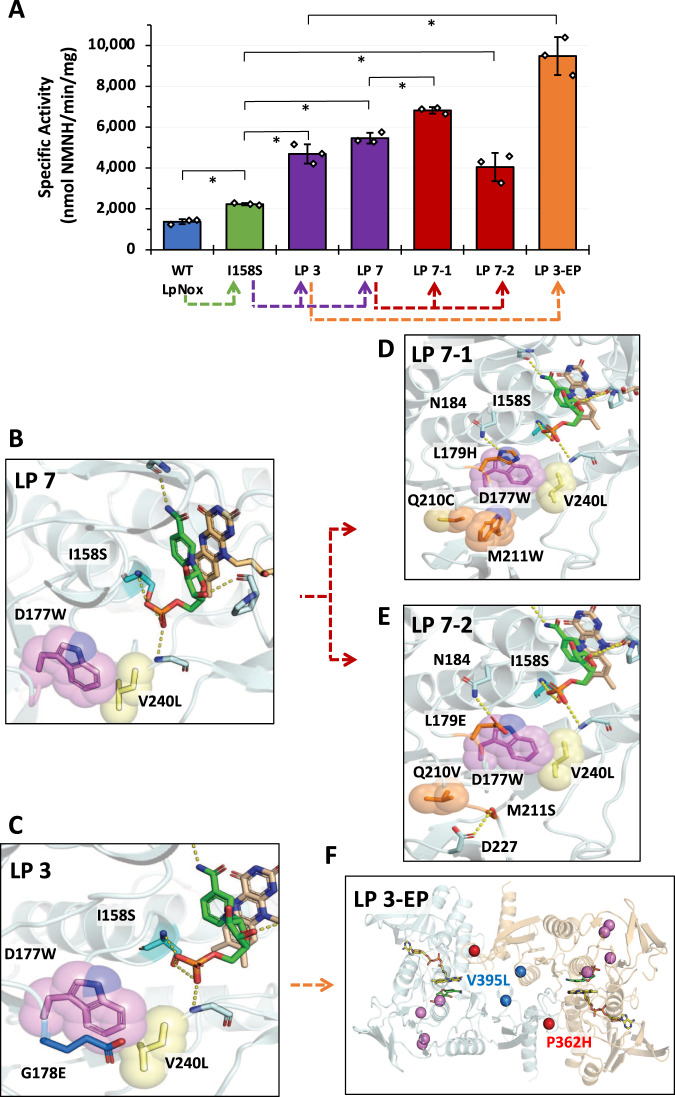
*Lp* Nox variants discovered through growth selection. **A** Enzyme specific activities with NMNH. Wild-type *Lp* Nox (blue) showed an initial level of activity that was increased ~2-fold with rational design of I158S (green). Growth selection of combinatorial mutants generated through the first round (purple) and second round (red) of site-saturation mutagenesis at the cofactor binding site and the second sphere, respectively, led to LP 3, LP 7, and LP 7-1, LP 7-2. A final step of random mutagenesis with error-prone PCR led to LP 3-EP (orange) that displays ~10-fold increase in NMNH activity compared to the wild type. The dashed line under the x-axis indicates the parental variants and different selection strategies. For all samples, *n* = 3 biologically independent replicates. For statistics, WT to I158S, *p* = 0.00038; I158S to LP 3, *p* = 0.012; I158S to LP 7, *p* = 0.0023; LP 7 to LP 7-1, *p* = 0.0016; I158S to LP 7-2, *p* = 0.045; LP 3 to LP 3-EP, *p* = 0.0014. **B** Model of LP 7 binding pose with FAD (peach) and NMNH (green). I158S forms a polar contact with the NMNH phosphate, while D177W and V240L rigidly fill the binding cleft where the adenosine would typically occupy. **C** LP 3 carries an additional G178E compared to LP 7. D177W must rotate further toward the inside of the pocket to accommodate the bulkier G178E. **D**, **E** LP 7-1, and LP 7-2 carry second-sphere mutations that rigidify the flexible regions on the protein surface through denser packing and new polar interactions, which enhances the protein stability. **F** Superposition of *Lp* Nox homology model with quaternary assembly of *Lb* Nox (PDB: 5VN0). Mutation positions lining the cofactor pocket are colored violet. The substitutions identified from random mutagenesis P362H (red) and V395L (blue) are remote from the active site and have an indirect effect on cofactor binding. Their surface exposure may affect multimeric assembly in addition to altering function of each individual monomer. Data are presented as mean ± standard deviation. The statistical significance was determined by two-tail *t*-tests (*: *p* < 0.05). Source data is provided as a Source Data file.

We have previously demonstrated that endogenously biosynthesized NMN^+^ can support GDH Ortho function and sustain cell growth, albeit to a much lower extent compared to exogenous NMN^+^ supplementation^8^. To promote healthier cell growth, we generated strain MX503 which features two primary modifications: (1) the chromosomal integration of *Ft* NadEV (Fig. 1B) and (2) a reduction in the total number of plasmids required to enable growth (Supplementary Table 2). Co-transformation of the supportive plasmid pLS503, carrying the genes encoding GDH Ortho and *Zm* Glf, and the redox partner panel plasmid, yielded a growth profile that mirrored the trend observed in MX502 without the need for supplementation of NMN^+^ in the selection media (Fig. 1E). As before, increasing NMNH specific activity from WT XenA to XenA D116E correlated with an improved capacity for growth, while the expression of the NADH and NADPH oxidase did not facilitate growth. Increased size difference between colonies carrying XenA WT and D116E suggests lower NMN^+^ availability increased the sensitivity of the selection (Fig. 1E, Supplementary Fig. 2).

Notably, even when the growth was considered rescued by the presence of XenA and XenA D116E, the selection strains still grow at a much lower rate than wild-type *E. coli* (Supplementary Fig. 3). This may originate from the disruption of the heavily utilized EMP glycolysis, which cannot be fully replaced by the ED pathway that naturally sustains a much lower carbon flux due to *E. coli’*s regulatory control^32^. Therefore, if the glucose uptake capacity through ED pathway can be enhanced through metabolic engineering or adaptive laboratory evolution^33^, superior NMNH-oxidizing enzymes may support even faster growth allowing them to be more readily and reliably selected.

### Engineering *Lp* Nox to utilize NMNH by rational design

With the selection systems in hand, we next sought to develop *Lp* Nox as an efficient NMNH-recycling catalyst. Wild-type *Lp* Nox was previously shown to have a basal level of promiscuous activity toward noncanonical cofactors^14,26^, which may indicate the propensity for this enzyme to receive a variety of NCBs, once engineered. The first round of rational design was based on the structural analysis of the cofactor binding pose. We tested a total of 15 single mutations at 8 sites with the potential to form novel polar interactions, particularly with the phosphate on the NMNH tail, or reduce NAD(P)H activity by reorganizing the cofactor binding pocket (Supplementary Fig. 4A). The detailed rationale for their design can be found in the Supplementary Information. We obtained a single mutant I158S with improved specific activity for NMNH by ~2-fold compared to wild type (Fig. 2A, Supplementary Fig. 4A), which serves as the starting point for subsequent engineering efforts. We hypothesized that the I158S side chain forms a hydrogen bond with the NMNH phosphate, which is necessary to anchor NMNH and opens opportunities for further design. This design strategy has been demonstrated in our previous work^8^. This test also shed light on the choice of sites that will be subjected to subsequent semi-rational engineering (Supplementary Fig. 4B).

### Improving NMNH activity by targeting the first-sphere residues

Based on this initial variant I158S, we sought to leverage the high-throughput growth selection platform developed to further improve NMNH activity by site-saturation mutagenesis. In this round, we targeted the first-sphere positions.^34^ These residues line the cofactor binding pocket and are analogous to the key residues that enabled our previously engineered GDH to utilize NMN^+^^8^ (Supplementary Fig. 5). We sampled D177, G178, and V240 with NNK codons and fixed I158S.

The resulting NNK library (pLS506) was introduced into the selection strain MX502, yielding ~2.4 × 10^6^ independent transformants, which was sufficient to cover >10-fold of the theoretical library size of 20^3^ = 8000. Selection was performed on agar plates with 20 g/L D-glucose in M9 minimal medium supplemented with 5 mM NMN^+^ at 30 °C. After incubation for 10 days, many colonies had formed on the selection plates. After subsequent colony isolation, ten were chosen for further analysis based on their fast growth, relative to other candidates.

Interestingly, sequencing of the ten candidates revealed a strong consensus for bulky or hydrophobic residues at both sites 177 and 240 (Supplementary Fig. 6, Supplementary Table 3). We chose variant LP 7 (I158S-D177W-V240L, Fig. 2B) to investigate further, because its mutation pattern is shared by three independently selected variants with different codons (Supplementary Table 3) suggesting strong selective pressure for this variant. We also chose LP 3 (I158S-D177W-G178E-V240L) because of its high similarity to LP 7, with just one additional mutation (Fig. 2C). Variants LP 3 and LP 7 exhibited ~4 and ~5-fold increase of specific activity toward NMNH, respectively, compared to WT *Lp* Nox (Fig. 2A).

We characterized the kinetic parameters of *Lp* Nox variants (Table 1) to investigate the structural basis for their improved activity. Wild-type *Lp* Nox displayed a small amount of initial specific activity for NMNH, with a catalytic efficiency of 2.3 ± 0.1 mM^−1^ s^−1^ with the linearized Michaelis-Menten equation (Fig. 2A, Table 1).

**Table 1:** Apparent kinetics parameters of *Lp* Nox variants

Enzyme	Cofactor	K_m_ (mM)	k_cat_ (s^-1^)	k_cat_/K_m_ (mM^-1^s^-1^)
WT Lp Nox (wild type)	NADH	0.025 ± 0.001	21 ± 0.3	840 ± 10
	NADPH	1.9 ± 0.2	18 ± 2	9.3 ± 0.2
	NMNH	ND^a^	ND^a^	2.3 ± 0.1
LP 7 (I158S-D177W-V240L)	NADH	1.7 ± 0.2	47 ± 4	28 ± 1
	NADPH	0.89 ± 0.04	27 ± 2	31 ± 1
	NMNH	ND^a^	ND^a^	18 ± 1
LP 3 (I158S-D177W-G178E-V240L)	NADH	1.9 ± 0.04	31 ± 1	17 ± 0.2
	NADPH	1.1 ± 0.1	28 ± 1	26 ± 1
	NMNH	ND^a^	ND^a^	10 ± 0.2
LP 3-EP (I158S-D177W-G178E-V240L-P362H-V395L)	NADH	ND^b^	ND^b^	49 ± 2^c^
	NADPH	ND^b^	ND^b^	51 ± 4^c^
	NMNH	ND^a^	ND^a^	23 ± 1

Reactions were performed in 50 mM Tris-HCl buffer pH 7.0 at 37°C with varied cofactor concentration. Replicates size for k_cat_/K_m_ of WT, LP 7 and LP 3-EP, *n* = 3. For other parameters, replications size n = 2. All data are reported as mean ± standard deviation. ND, not determined. Source data is provided as a Source Data file

^a^Enzyme could not be saturated with the cofactor concentrations tested

^b^Enzyme exhibited inhibition by the cofactor that cannot be fit with the standard substrate inhibition rate equation

^c^Estimated based on kinetics under lower cofactor concentrations before the inhibition was observed

LP 7 contains D177W-V240L which are modeled to densely pack against each other and fill the void typically occupied by the AMP moiety in NADH (Fig. 2B). Characterization of variants missing either one of the D177W-V240L duet supports their predicted synergy (Supplementary Fig. 7). This model suggests that the larger NADH is excluded from establishing its native binding pose through steric clash, while the smaller NMNH is minimally affected by the additional bulk. The mutation D177W also results in the loss of key polar contacts to the NADH, causing the K_m_ value for NADH to increase from 0.024 ± 0.005 mM in the wild type to 1.7 ± 0.2 mM with LP 7. Furthermore, the added steric hindrance may restrict the flexibility of nearby loops and result in greater pre-organization of the active site (vide infra). The catalytic efficiency of LP 7 for NMNH increased ~8-fold from the wild type to 18 ± 1 mM^−1^ s^−1^ (Table 1).

LP 3 shares the core I158S-D177W-V240L mutations as LP 7, and LP 3 additionally carries G178E. The increase in side chain size at the 178 position likely causes diminished loop flexibility and the larger glutamate slides underneath D177W to seal the tryptophan mutation in the adenosine cleft (Fig. 2C). As a result, LP 3 features further diminished binding affinity for both NADH and NADPH with K_m_ values of 1.9 ± 0.04 mM and 1.1 ± 0.1 mM, respectively (Table 1).

### Restoring protein stability by targeting second-sphere residues

We sought to further improve LP 7 by targeting residues L179, Q210, and M211 through another round of site-saturated mutagenesis and growth selection. These sites were suggested by Cofactor Specificity Reversal-Structural Analysis and Library Design (CSR-SALAD)^35^, a bioinformatics tool that facilitates the automated design of cofactor specificity switch between NAD(H) and NADP(H). We hypothesized that the predicted, activity-boosting effects of these three sites would be translatable to engineering NMNH activity, since they are second-sphere residues^34^ that do not directly contact the cofactor but may modulate the activity allosterically.

**Fig. 3:**
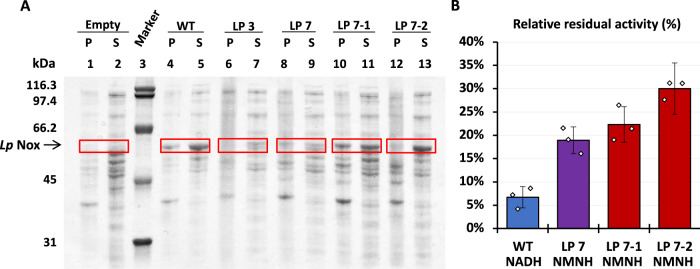
Variants selected from the second round of growth selection display increased solubility and thermostability. **A** SDS -PAGE analysis of protein solubility. The insoluble pellet (P) and supernatant (S) fractions obtained through centrifugation following cell lysis are evaluated for Nox expression level. Variant LP 7-1 and LP 7-2 show increased solubility fraction (lane 10/11 and 12/13) compared to LP 7 (lane 8/9). WT *Lp* Nox (lane 4/5) and empty plasmid harboring samples (lane 1/2) serve as controls. **B** Relative residual activity before and after heat treatment. After 23 h of heat treatment at 50 °C, WT (blue) activity with native cofactor was diminished to 7% ± 2 %. Meanwhile engineered variants LP 7 (purple), LP 7-1 (red), and LP 7-2 (red) show enhanced thermal stability. Moreover, LP 7-2 (30% ± 6%) displays significantly higher thermal stability than LP 7 (19% ± 3%). Data are presented as the ratio between the average of three biologically independent replicates ± error. Scattered data points represent the ratio between individual samples after heat treatment and the averaged measurement before treatment. Error in relative residual activity is calculated by propagation of the error in treated and untreated samples’ measurement. Source data is provided as a Source Data file.

A site-saturated mutagenesis library (pLS509) with the NNK codon using LP 7 as the template was constructed and introduced in strain MX503. Growth-based selection was conducted similarly to the last round described above, with the modification that no exogenously supplied NMN^+^ was added to the selection plates when we switched to the self-sufficient selection strain. After colony isolation, we characterized the two fastest-growing variants, LP 7-1 and LP 7-2 (Fig. 2A, D, E, 3).

LP 7-1 represents a further improvement to LP 7 for specific activity, from ~5500 to ~6600 nmol NMNH min^−1^ mg^−1^ (Fig. 2A). Importantly, both LP 7-1 and LP 7-2 showed markedly improved stability compared to their precursor LP 7, as evident by their substantially higher expression levels as soluble proteins at a comparable level to WT *Lp* Nox (Fig. 3A). Additionally, LP 7-2 exhibited robust thermal stability with ~30% residual activity remaining after 23 h of heat treatment at 50 °C, which is significantly higher than its parent variant LP 7 (~19% residual activity) (Fig. 3B).

LP 7-1 (L179H-Q210C-M211W) and LP 7-2 (L179E-Q210V-M211S) share the same mutations as LP 7 with additional mutations to positions 179, 210 and 211. Rosetta modeling suggests that the additional mutations improve protein folding fitness by rigidifying flexible regions on the protein surface^36^. The mutation L179H in LP 7-1 is predicted to form a hydrogen bond with N184, which fastens the loop that L179H resides (Fig. 2D). L179E in LP 7-2 is predicted to have the same mode of action (Fig. 2E). In LP 7-1, M211W and Q210C are suggested to decrease this region’s backbone flexibility through the added bulk and hydrophobic interaction (Fig. 2D). In LP 7-2, a similar effect may be mediated by the new hydrogen bond between M211S and the nearby D227 (Fig. 2E).

The new variants’ ease of soluble expression and the enhanced thermal stability will translate to lower cost in large-scale industrial applications. Furthermore, by restoring the engineered enzyme’s stability, these new mutations may unlock evolutionary trajectories that involve more fitness-taxing mutations in future engineering^37–39^.

### Random mutagenesis identified beneficial mutations more distal from active sites

In parallel to targeting the second sphere, we used error-prone PCR to search a broader protein sequence space, aiming to improve LP 3. Error-prone PCR mutagenesis was performed on template LP 3. A mutation frequency of two amino acid changes per construction was targeted. The library was introduced into selection strain MX502, yielding ~2.14 × 10^6^ independent transformants. Matching the growth-based selection stated previously, the error-prone PCR library was subjected to selection on media containing 0.5 mM NMN^+^. After 11 days of monitoring, colonies growing faster than the template, indicating improved activity of cofactor cycling, were isolated. One variant LP 3-EP (I158S-D177W-G178E-V240L-P362H-V395L) was identified to exhibit an ~8-fold increase in NMNH-dependent specific activity compared to the wild-type (Fig. 2A).

LP 3-EP accumulates two more mutations compared to LP 3, namely P362H and V395L (Fig. 2F). The mutations P362H and V395L appear distal to the active site, ~30 Å and ~20 Å away, respectively; there is no clear rationale for their beneficial effects from the static homology models as they hang from surface regions and do not dramatically alter hydrophobic or electrostatic interactions. Overlay of the model on the biological assembly of *Lb* Nox (PDB: 5VN0) indicates that P362H and V395L are both positioned near the subunit interface, and they may affect cross-monomer interactions (Fig. 2F). Notably, the activity of LP 3-EP is strongly inhibited above 1 mM NADH or NADPH. However, this kinetic behavior fits poorly with the well-established substrate inhibition rate equation^40,41^, suggesting that NAD(P)H may exert complex effects on this enzyme, such as excessive uncoupling which generates reactive oxygen species and damages the enzyme. Interestingly, this inhibition effect was not seen when NMNH was utilized, which may indicate better coordinated catalysis for this cofactor. LP 3-EP’s catalytic efficiency for NMNH was 23 ± 1 mM^−1^ s^−1^, a ~10-fold increase from the wild type and ~2.5-fold gain from LP 3 (Table 1).

### Engineered *Lp* Nox showed increased activity with other noncanonical cofactors

After demonstrating *Lp* Nox variants have increased activity towards our targeted noncanonical cofactor, NMNH, we tested them with additional NCBs, MNAH and BNAH. These simpler noncanonical redox partners are considered more applicable in large scale thanks to their lower synthesis costs (Supplementary Table 1)^4,42^. In addition, by analyzing activity patterns toward structurally distinct cofactor analogs, we can further pinpoint the mechanism of cofactor-protein interaction in engineered *Lp* Nox.

**Fig. 4:**
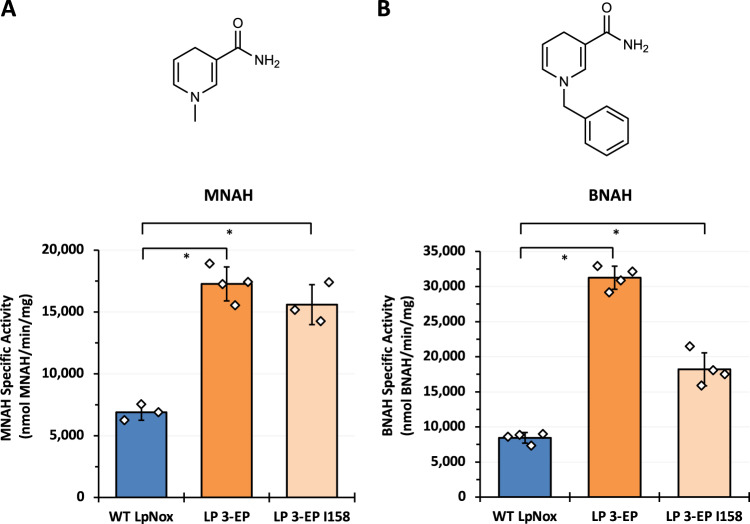
Enzymatic specific activities of engineered *Lp* Nox variants towards other biomimetic cofactors. **A** Compared to WT *Lp* Nox (blue), engineered *Lp* Nox variant, LP 3-EP (orange), displayed ~2.5-fold higher activity towards MNAH. Without the I158S mutation to form the hydrogen bond with the phosphate, LP 3-EP I158 (beige) still shows similar MNAH activity suggesting that the remaining mutations function allosterically since they should not have direct contact with the binding pose of MNAH. For LP 3-EP, *n* = 4 biologically independent replicates. For other samples, *n* = 3 biologically independent replicates. For statistics, WT to LP 3-EP, *p* = 0.000074; WT to LP 3-EP I158, *p* = 0.00097. **B** Similar to the activity trend with MNAH, *Lp* Nox variant, LP 3-EP (orange), shows a ~4-fold increase in specific activity compared to WT *Lp* Nox (blue) using BNAH. Furthermore, LP 3-EP I158 (beige) still shows higher activity with BNAH than WT *Lp* Nox. For all samples, *n* = 4 biologically independent replicates. For statistics, WT to LP 3-EP, *p* = 2.5 ×10^-7^; WT to LP 3-EP I158, *p* = 0.0014. Data are presented as mean ± standard deviation. The statistical significance was determined by two-tail *t*-tests, (*: *p* < 0.05). Source data is provided as a Source Data file.

The most active *Lp* Nox variant, LP 3-EP, was tested for activity using MNAH and BNAH at 0.3 mM. LP 3-EP showed ~2.5 and ~4-fold higher specific activity toward MNAH and BNAH, respectively, compared to WT *Lp* Nox (Fig. 4A, B). Since the I158S mutation in LP 3-EP was originally designed to form a hydrogen bond with the NMNH phosphate, we hypothesized that this mutation might not be required for interacting with MNAH and BNAH, which have non-polar moieties attached to the nicotinamide. When the 158 position is reverted to isoleucine (pLS513), the resulting variant LP 3-EP I158 still retains higher specific activities compared to the wild-type enzyme, especially for MNAH (Fig. 4A). This is particularly interesting because none of the remaining mutations in LP 3-EP I158 are predicted to directly contact MNAH given the cofactor’s small size. Therefore, we postulated that these mutations may affect the ensemble of conformations sampled by the enzyme, and we performed molecular dynamics simulations to understand this effect.

### The effects of mutations on *Lp* Nox structural flexibility

Based on the location and crowding of the D177W-G178E-V240L mutations, we hypothesized that this group of residues, beyond simply reshaping the binding interface, would have an additional effect of increasing the rigidity of the active site. A stiff active site has been associated with increased catalytic turnover based on the principle that pre-organized residues can immediately latch onto the ligand and initiate catalysis^43^. Active sites with a high degree of flexibility may result in frustrated binding events where the ligand encounters the protein, but the protein cannot achieve the necessary intermediate state for the reaction as the catalytic residues excessively sample irrelevant poses.

**Fig. 5:**
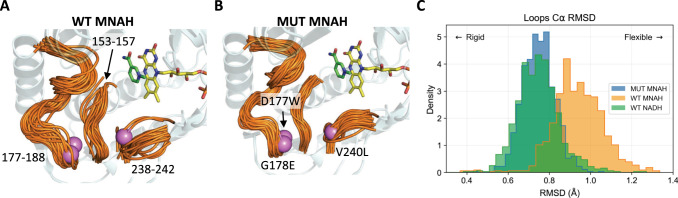
Comparison of Rossman fold loop flexibility between wild type and engineered *Lp* Nox. 40 random frames are selected and aligned from each simulation to visualize the range of conformations sampled. The loop regions measured are highlighted in orange (residues 153-157, 177-188, and 238-242) and envelope the AMP portion of the native NADH cofactor. The three mutated positions in the binding site (D177W, G178E, and V240L) are colored as violet spheres. **A** WT with MNAH bound displays high flexibility as indicated by large displacement of the strands. The fluctuations impede organization of the active site to support cofactor binding and catalysis. **B** Mut (LP 3-EP I158) with MNAH bound shows more tightly packed bundles for the overlaid structures, demonstrating greater rigidity compared to the WT. The addition of the large, hydrophobic substitutions crowds the AMP binding cleft and reduces the volume of space for the loops to explore alternative conformations. **C** Quantification of the loop flexibility through alpha carbon RMSD illustrates that WT with MNAH exhibits higher loop flexibility with 0.94 ± 0.13 Å in comparison to Mut with MNAH at 0.75 ± 0.08 Å or WT with NADH at 0.74 ± 0.10 Å.

We performed 400 ns molecular dynamics (MD) simulations with 2 replicates for the 3 conditions: *Lp* Nox wild type (WT) with NADH, WT with MNAH, and LP 3-EP I158 (Mut) with MNAH. The rigidity of the active site loops surrounding the AMP pocket was compared through RMSD alignment to the starting homology model (Fig. 5). Overlay of 40 randomly sampled frames from the WT with MNAH and Mut with MNAH condition illustrate how the WT protein experiences much greater plasticity when bound to this smaller cofactor with the loops having wider spread (Fig. 5A). On the other hand, the overlay of loops in the Mut model bundle more tightly, indicating greater stability (Fig. 5B). The differences in flexibility are quantified through comparison of the RMSD distributions for the highlighted loop residues after alignment to the starting pose (Fig. 5C). Mut with MNAH bound had loops RMSD of 0.75 ± 0.08 Å, nearly identical to the value found for the WT with native cofactor NADH bound of 0.74 ± 0.10 Å. The WT with MNAH bound showed greater active site loop flexibility with 0.94 ± 0.13 Å RMSD and prominent rightward shift in the histogram of RMSD values. The presence of the bulky residues introduced by the mutations possibly mimic the effect of having the larger NADH bound, reducing the conformational entropy of the loops surrounding the AMP pocket.

### Correlation between conformational dynamics and catalytic activity

**Fig. 6:**
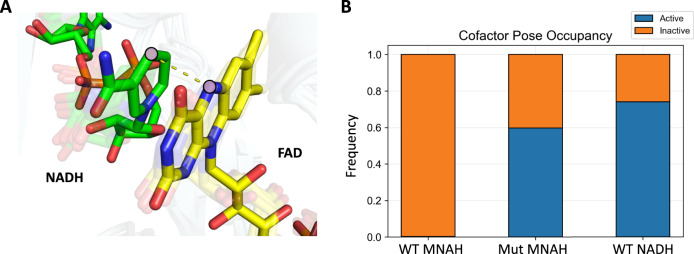
Measurement of hydride transfer efficiency of wild type and engineered *Lp* Nox. **A** Electrons are shuttled from the nicotinamide cofactor (green) to FAD (yellow) through movement between the nicotinamide ring C4 and FAD N5. A catalytically competent enzyme must be able to support this contact, otherwise catalysis cannot occur. A threshold of 4.5 Å is bounded to categorize binding poses below as being active and poses with distances above as being inactive since the cofactors are too far to engage in hydride transfer. **B** WT with MNAH bound samples the active pose with only 1% frequency. The inability to maintain the MNAH within hydride transfer distance is resolved in the Mut (LP 3-EP I158) which samples the active pose at 60% frequency. The WT *Lp* Nox firmly anchors the native NADH through the AMP handle and occupies the active pose in 74% of the frames.

**Fig. 7:**
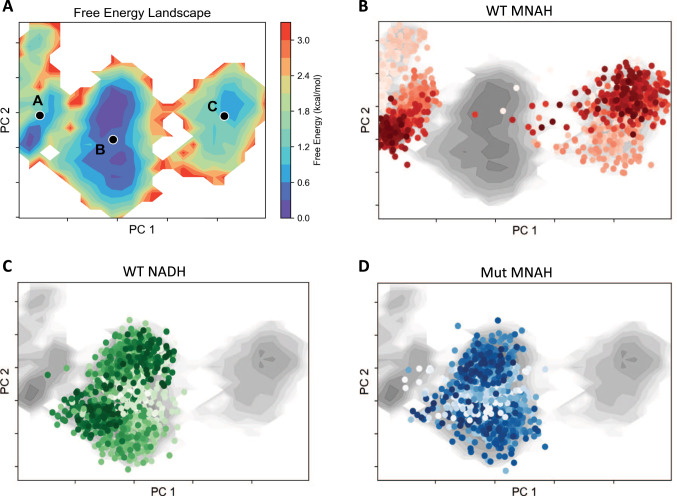
Comparison of conformational landscape of wild type and engineered *Lp* Nox. Global variation in backbone ensembles is represented by PCA dimensionality reduction of alpha carbon dihedrals, all samples are combined onto shared latent space and plotted on the first two principal components. Color intensity in panels B, C, and D indicate temporality, with lighter colors signifying conformations at the start of the simulation, and darker colors marking samples near the end. **A** The combined free energy landscape shows grouping into three local minima, whose centers are labeled **A, B,** and **C** through K-Means clustering. **B** WT with MNAH samples disjoint regions of conformation space along the first principal component corresponding to clusters A and C, and rarely traverses through the central cluster B. The replicates do not overlap, suggesting a high degree of flexibility and divergence in conformations. **C** Conformations of WT with NADH are focused on cluster B which is spread more broadly along the second principal component. **D** Mut (LP 3-EP I158) with MNAH occupies cluster B and does not stray into other regions, similar to WT with NADH. The Mut with MNAH surveys a comparable ensemble of backbone conformations that are characterized by catalytic ability to the WT with NADH.

To capture the effects of altered structural flexibility in the engineered mutant on catalysis, we compare both localized effects by measuring hydride transfer propensity between the nicotinamide and FAD (Fig. 6), and global conformational dynamics (Fig. 7). Nox-catalyzed reactions are mediated by a FAD cofactor that is proposed to accept hydride transfer from NADH to become FADH_2_, which then passes its electrons to oxygen. Hydride transfer occurs between the nicotinamide C4 and FAD N5 (Fig. 6A), this distance is observed to be ~3.1 Å in the crystal structure of the related *Lb* Nox with NADH bound (PDB: 5VN0). Inactive enzymes will rarely sample the necessary catalytic geometry, resulting in no reaction; conversely, highly active enzymes must support close contact between the nicotinamide and FAD. We defined a threshold of 4.5 Å as the maximum distance between the nicotinamide C4 and FAD N5 for active catalysis since separation greater than 4.5 Å typically meant that the nicotinamide cofactor had exited the binding cavity during the MD simulation and binned the hydride transfer distances into active and inactive categories.

The distribution of cofactor poses correlates with the different levels of activity observed. WT with NADH bound maintained the active geometry for hydride transfer in 74% of the frames, and only 1% of the frames with MNAH bound (Fig. 6B). Visual inspection of the WT trajectories suggests that the measured high loop flexibility when binding to MNAH (Fig. 5) resulted in rattling and bumping of the cofactor out of the binding pocket, and furthermore, impeded the cofactor from returning. Mut (LP 3-EP I158) with MNAH displayed 60% occupancy for the active pose supporting hydride transfer (Fig. 6B). These observations suggest that dense hydrophobic packing from the D177W-G178E-V240L mutations served to lock the mobility of the binding pocket loops and allowed the MNAH to bind with reduced interference.

Enzymes sample an ensemble of backbone configurations with varying functional capabilities. Enzyme variants with enhanced activity have been discovered to sample pre-existing catalytically active states with greater frequency^44,45^. Based on this observation, we hypothesize that the discovered mutations may function by shifting the population of conformation states toward those more relevant to catalysis.

The free energy landscapes of the enzyme systems are visualized through PCA dimensionality reduction to resolve coordinates of maximal variance in the alpha-carbon dihedrals over the course of the simulations (Fig. 7). Combined data from all MD trajectories is projected onto shared latent space defined by the first two principal components and cluster into three distinct groups. These groups each capture different local minima and are labeled by K-Means clustering as Groups A, B, and C (Fig. 7A).

Each data point represents a state, and each point is shaded by temporality with lighter colors indicating those at the start of the simulation and darker points representing data near the end. WT with MNAH occupies Groups A and C, while hardly crossing into Group B (Fig. 7B). Most notably there is little overlap with Group B where the bulk of catalytically active states are found as indicated by the simulation of WT with NADH (Fig. 7C). Mut (LP 3-EP I158) with MNAH shows similar population distribution to WT with NADH by clustering in Group B (Fig. 7D), indicating the mutations reshape the conformational landscape of *Lp* Nox with MNAH bound to resemble that of the WT when using its native cofactor NADH.

## Discussion

We have established a robust, high-throughput selection platform with tunable selection stringency for NMNH-utilizing enzymes. This was enabled by the optimization of *E. coli* cells that require an NMN^+^-based glycolytic pathway to grow. Notably, the selection has been shown to report the NMNH-utilizing activity of XenA and Nox, two distinct enzymes, which suggests universal compatibility in engineering various target enzymes. The Nox variants obtained from the growth selection carry first-sphere mutations which are cooperative and have a minimal or deleterious effect individually (Fig. 2B, C; Supplementary Fig. 7), second-sphere mutations that restore the soluble expression level of engineered enzymes back to that of the wild-type (Fig. 2D, E, 3) and are difficult to rationally design due to the complexity of protein folding, as well as random mutations that are even more distal to the active site (Fig. 2F). These variants arguably cannot be discovered without a rapid and high-throughput method that enables the deep search of protein sequence space. Previous efforts to engineer proteins for NCB binding have not led to clear design principles as few experimental structures with the NCB bound have been solved, and only a small fraction of the work has been devoted to understanding the mechanisms behind the improved activity in engineered variants.

Nowak et al.^46^ mutated *Sulfolobus solfataricus* glucose dehydrogenase (*Ss* GDH) to have activity with NCBs such as 3-carbomyl-1-phenethylpyrindin1-ium chloride (P2NA^+^) with unclear mechanism. Our design strategy differs from Nowak et al.^26^ in that we target more transferrable mutations near the conserved dinucleotide binding domain of the Rossman fold, while they aimed to restructure binding near the site of catalysis at the nicotinamide ring. In another study, Huang et al.^16^ screened variant libraries for NMN^+^ active *Thermotoga maritima (T. maritima)* 6-phosphogluconate dehydrogenase (6PGDH), resulting in the Mut 6-1 variant with ~50-fold increase in NMN^+^ catalytic efficiency and promiscuous activity with other NCBs. However, there is no clear explanation for the contribution of these accumulated mutations distributed throughout the structure. The lack of clear structural basis for improved activities can be aided with MD analysis. Liu et al.^47^ performed virtual screening with computational binding affinity predictions and MD analysis of binding pose stability to select mutant P450-BM3 using NMNH. Their pipeline led to identification of P450-BM3 S848R with ~2-fold increase in NMNH activity over the wild type. Nevertheless, the success rate of their approach was 10%, highlighting the need for more reliable simulation methods and opportunity to increase sample efficiency by coupling computational predictions with high-throughput screens.

In this study, we investigate cofactor binding at the molecular level through directed evolution and computational simulations and harmonize the results of previous engineering efforts into accessible design guidelines. (1) Mutations in the first sphere of the cofactor binding pocket can be readily interpreted through their effects on hydrophobic packing or polar interactions with the ligand. I158S falls in this category, and its parallel appearance in *Ss* GDH I192T-V306I^46^ raises the importance of positioning the Rossman pyrophosphate binding alpha helix to coordinate cofactor binding. (2) Through growth selection, we deeply explored combinatorial mutations in the adenosine pocket and discovered LP 3 and LP 7 that shared dense hydrophobic packing with D177W-V240L in the vacant cleft that the larger NADH would occupy. Similar sets of mutations were found in previous work by Huang et al. where Mut 3-1 6PGDH^16^ seeded later substitutions dispersed through the protein. Although it is not clear from a static model, we showed the benefit of padding the interior of the adenine cavity to favorably modulate protein conformational dynamics and maintain cofactor contact with FAD.

The growth selection demonstrated here is well suited for developing NMN(H)-dependent enzymes that optimally function in vivo. If integrated into the engineered hosts which biosynthesize NMN^+^^8,12,28–31^, these enzymes are promising catalysts for whole-cell biotransformation. Particularly in *E. coli*, a three-pronged central carbon metabolism can be envisioned where the EMP pathway produces NADH, the PP pathway NADPH, and the most energetically favorable but natively less-utilized ED pathway is re-purposed to produce NMNH^32^. This design allows reducing power to be partitioned toward orthogonal metabolic pathways directly at the source and then protected from the numerous native NAD(P)H-consuming pathways. Based on this blueprint, the ED pathway needs to be reinvigorated in *E. coli* to increase its flux capacity^48,49^. The tradeoff between cell fitness and production yield is significant when the central metabolism is rewired, for example to bias NADPH generation to power the desired pathways^50,51^. Therefore, the balance between NMNH-generating ED pathway and the other two branches also must be tuned, which can be guided by systems studies and flux balance analysis.

## Methods

### Media and growth conditions

Cloning was carried out with *E. coli* XL-1 Blue (Supplementary Table 2). All *E. coli* were cultured in 2x YT medium containing 16 g/L tryptone, 10 g/L yeast extract, and 5 g/L NaCl, unless otherwise noted. M9 Wash Buffer consisted of 1 mM MgSO_4_, 0.1 mM CaCl_2_, trace metal mix A5 with Co (Sigma Aldrich), and BD Difco M9 salts (Fisher Scientific). M9 Selection Media contained M9 Wash Buffer with the inclusion of 20 g/L D-glucose, 1 mM nicotinamide, 0.1 mM IPTG, and 0.05% (w/v) arabinose, 200 mg/L ampicillin, 50 mg/L for kanamycin, and 50 mg/L for spectinomycin. For solid media M9 Selection Plates, 15 g/L agar was added to M9 Selection Media. Expression media consisted of 2x YT media supplemented with 20 g/L D-glucose, 1 mM nicotinamide, and antibiotics as above. NMN^+^ was added to a concentration of 0, 0.5, 2, or 5 mM to M9 Selection Media as needed and specific concentrations are noted in the selection experiment methods below. All strains were cultured at 37 °C with 250 r.p.m. agitation and concentrations for antibiotic selection were 100 mg/L for ampicillin, 50 mg/L for kanamycin, and 50 mg/L for spectinomycin, unless otherwise noted.

### Strain construction

Strain MX501 was generated by eliminating kanamycin resistance carried by *E. coli* strain MX103^8^, and MX501 served as the template strain for MX502 and MX503. Plasmid pCP20 was used to eliminate kanamycin resistance between rounds of genomic integration or knockouts^52^.

Strain MX502 was constructed by introducing plasmids pLM106 and pLS502 into strain MX501. A single colony was used to inoculate a 4 mL 2x YT overnight culture. To generate dense electro-competent cells of *E. coli* MX502 for control experiments and for library transformations, cells were cultured in 200 mL SOB medium at 30 °C with shaking at 250 r.p.m. until OD_600nm_ reached 0.4–0.6. The culture was chilled on ice for 15 minutes and the cells were pelleted at 4 °C, 4000×g. The cells were washed at 4 °C three times with 40 mL sterile 10% glycerol in water. After, cells were resuspended with 500 µL sterile, ice-cold 10% glycerol, aliquoted, and stored at –80 °C.

Strain MX503 was constructed by chromosomal integration of the P_lacO1_ *Ft* NadEV operon using the lambda red homologous recombination plasmid pKD46^52^. The integration cassette was constructed by amplifying the operon from plasmid pSM103^8^ from the *P*_LlacO1_ promoter to the stop codon of *Ft* NadV. Splicing-by-overlap-extension PCR was used to generate and amplify a fragment carrying a kanamycin cassette adjacent to the P_lacO1_ *Ft* NadEV cassette using KOD Xtreme Hot Start DNA Polymerase (Novagen). Homologous arms (1000 bp) of the kanamycin- *P*_LlacO1_-*Ft* NadEV expression cassette were designed for integration within the site of non-essential gene pyruvate oxidase. Integration was confirmed by colony PCR and sequencing of the region.

### Plasmid construction

All PCR fragments were generated using PrimeSTAR Max DNA Polymerase (TaKaRa) unless otherwise noted. PCR reactions were purified by gel electrophoresis and were assembled into the pQElac vector backbone (Supplementary Table 2) using Gibson isothermal DNA assembly method^52^, unless otherwise noted.

Plasmid pLS502 was constructed using plasmid pSM103^8^ as a template to amplify *Ft* NadEV*-Zm glf* gene, which is inserted subsequently into pZAlac vector backbone (p15A *ori*, Kan^r^). Plasmid pLS503 was constructed using pSM103 and pSM106^8^ as templates. RBS - *Zm glf* insert was generated by pairing a forward primer binding at the RBS (ribosomal binding site) adjacent to *Zm glf* on pSM103 and a reverse primer binding at the end of *Zm glf* using pSM103 as the template. RSF - *Bs* GDH I195R-A93K-Y39Q-S17E, (RSF *ori*, Spec^r^) backbone was generated by pairing a reverse primer binding at the end of *Bs* GDH and a forward primer binding at the terminator region following the end of *Bs* GDH using pSM106 as the template. These fragments were assembled to form pLS503.

### Protein expression and purification

*Lp* Nox variants were expressed with *E. coli* BL21 (DE3). Expression was carried out by inoculation of 20 mL 2x YT media supplied with antibiotics. Cells were induced with 0.5 mM IPTG at an OD_600nm_ of 0.6–1.0 and incubated for 24 h at 27 °C with shaking. After expression, cells were pelleted and resuspended in Zymo His-Binding buffer (Zymo Research Corporation) supplemented with 0.15 mM FAD, 1 μL/mL DNase (Sigma Aldrich), and 1 mM InSolution AEBSF (Sigma Aldrich). Cells were lysed by bead beating with 0.1 mm glass beads. Lysates were clarified by centrifugation at 4 °C, 20,000 xg,15 min. Protein purification was performed using His-Spin Protein Miniprep (Zymo Research Corporation) according to the manufacturer’s instructions. Protein concentration was determined by Bradford assay, using bovine serum albumin as a standard. Proteins were aliquoted and stored with 20% (v/v) glycerol at −80° C.

### Standard selection protocol of *Lp* Nox libraries and control plasmids into MX502

A total of 20 µL of library DNA was added to 200 µL electro-competent cells. Plasmids pEK102 (XenA WT) and pLS504 (*Lp* Nox WT) served as positive and negative controls, respectively. For each control, 1 µL of plasmid was added per 50 µL of cells. Immediately after electroporation, 600 µL of SOC media was used to transfer the solution to a microcentrifuge tube. Cells were then rescued at 37 °C with shaking for 1 hour.

After rescue, library cultures were transferred into 40 mL of Expression Media in a 250 mL baffled flask. Control cultures were transferred into 10 mL of expression media in loosely capped 50 mL conical tubes. Serial dilution of the library culture was plated on 2x YT agar plates with antibiotics to estimate transformation efficiency. The remaining cells were grown at 30 °C with shaking for 4 h before induction with 0.5% (w/v) arabinose and 0.1 mM IPTG, then expressed for 4 h before being collected by centrifugation. To prepare cells for the selection condition, 1 mL of each culture was pelleted and washed three times with 1 mL M9 Wash Buffer, described above.

### Selection of *Lp* Nox library pLS506 and pLS509

The washed library culture was diluted to a final concentration of ~10^5^ cells/mL in M9 Wash Buffer. 100 µL of this suspension was plated on M9 Selection Plates. Plates were incubated at 30 °C and monitored periodically for 10 days. After 10 days, colonies from each library plate and control plates were streaked onto fresh M9 Selection Plates to isolate variants and validate growth. For library pLS506 and pLS509, 14 out of the 30 and 7 out of the 24 re-steaked colonies grew significantly on the new plate compared to the controls and template variant. After 6 days, the plates containing the restreaked colonies were evaluated for growth. Colonies with diverse growth phenotypes were selected for sequencing and NMNH activity characterization. Colonies were cultured in 2x YT liquid media overnight, and plasmids were extracted using QIAprep Spin Miniprep kit (Qiagen) to yield plasmids pLS507, pLS508, pLS510 and pLS511.

### Error-prone library construction

The *Lp* Nox Error-Prone PCR Library was cloned using the GeneMorph II Random Mutagenesis Kit (Agilent) to generate approximately 2 random mutations per template on variant LP 3. A panel of starting template material (150 ng, 200 ng, 250 ng) was initially evaluated to tune the range of mutations. To construct the *Lp* Nox error-prone insert library, forward and reverse primers containing BamHI-HF and XbaI digestion site respectively were used. The gel-purified insert fragment and plasmid pLS504 (backbone) were separately digested with BamHI-HF and XbaI (NEB). Digested fragment with correct length were assembled by ligation, desalted, and transformated into the of ElectroMAX DH10β cells (Invitrogen) using electroporation. Cells were rescued with SOC at 37 °C for 1 h and added to 10 mL 2x YT medium with appropriate antibiotics. The error-prone library size was determined by serial dilution of culture on 2x YT agar plates with ampicillin. Six single colonies were randomly picked and cultured to extract plasmids. The sequencing results confirmed the average number of random mutations was 2.33 per variant. After performing the serial dilutions, the remaining liquid culture was incubated for at 37 °C with shaking. After 10 h, plasmids were extracted using the QIAprep Spin Miniprep kit, resulting in the pooled error prone library.

### Error-prone library selection of *Lp* Nox

Following standard selection protocol of *Lp* Nox stated above, the washed library culture was diluted to 10^6^ cells/mL and 10^5^ cells/mL. 50 µL of each concentration of suspension was plated on five M9 selection plates. Plates were incubated at 30 °C and monitored periodically for 11 days. Then, 59 single colonies from library plates and colonies from the control plates were streaked onto fresh M9 selection plates to isolate variants and validate growth. After 8 days, plate growth was evaluated following protocol described above in library pLS506 and pLS509 selection, discovering pLS512 (LP 3-EP).

### XenA activity assays

XenA specific activity assays were performed in a solution containing 200 mM potassium phosphate buffer at pH 7.5, 0.2 mM reduced redox cofactor, and 5 mM ketoisophorone at 37 °C. Purified XenA was spiked into the solution to initiate the reaction. Reaction progression was monitored by overserving the change in light absorption at 340 nm, corresponding to the rate of consumption of the reduced cofactor.

### NMNH generation

NMNH generation was performed in 20 mL reaction volumes in a 50 mL conical tube containing 200 mM Tris-Cl at pH 8.0, 100 mM sodium chloride, 3 mM potassium chloride, 200 mM glucose, and 20 mM NMN^+^. The reaction was initiating by spiking 500 ng of purified *Bacillus subtilis* glucose dehydrogenase I195R^8^. The reaction was incubated at 30 °C until reaction progression stopped, approximately 6 h. Reaction progression was monitored by observing the increase in light absorption at 340 nm, corresponding to the reduction of NMN^+^ to NMNH. Then, the solution was filtered through a 15 kD cutoff filter to remove the protein. To purify the NMNH, the flowthrough was mixed with HPLC-grade acetonitrile to a final concentration of 70% acetonitrile, and the solution was passed through a Phenomenex Strata NH2 (2 g / 12 mL, 55 µm, 70 Å) solid phase extraction column. The column was washed twice with 4 mL of 70:30 acetonitrile:water, then twice with 4 mL of 50:50 acetonitrile:water, twice with 4 mL of 30:70 acetonitrile:water, twice with 4 mL of nanopurified water, and finally twice with 4 mL of methanol. To elute the NMNH from the column, 2 mL of a solution containing 50:50 ammonium hydroxide:methanol was added to the column and incubated for 3 minutes before eluting. The elution step was repeated until no NMNH remained in the flow through, as monitored by light absorption at 340 nm. The elution fractions containing NMNH were pooled and aliquoted in 1 mL volumes into clean tubes. The solutions were dried over air and stored at −80 °C until use.

### *Lp* Nox enzymatic assays with NMNH, NAD(P)H

The *Lp* Nox-specific activity assays (Fig. 2A) were performed at 37 °C in 50 mM Tris-Cl buffer pH 7.0. The reactions were started by adding the purified protein to the prewarmed 37 °C assay mixture containing 0.3 mM of cofactor. The rate of light absorbance change at 340 nm was used to determine the rate consumption of NAD(P)H and NMNH using a SpectraMax M3 microplate reader. For apparent kinetics parameter determination (Table 1), cofactor concentrations were varied from 0 to 2 mM and the results were fitted to the standard Michaelis-Menten equation. When the cofactor concentration tested could not saturate the enzyme activity or during the presence of substrate inhibition, the Lineweaver-Burk equation was used to determine catalytic efficiencies (k_cat_/K_m_). Cofactor concentrations over 2 mM could not be measured accurately on spectrophotometer due to detector saturation.

### *Lp* Nox variants’ thermostability characterization

Thermostability (Fig. 3B) was tested by incubating the enzyme at 50 °C for 23 h and measuring residual enzymatic activity relative to each sample’s initial measurement following the protocol descried above. Proteins were cooled down to assay temperature, 37 °C, from 50 °C before initializing the reaction.

### Protein solubility analysis

WT *Lp* Nox, variants, and empty plasmid^8^ (pSM105) were expressed following the Protein Expression and Purification protocol. Cell pellets were resuspended to an OD_600nm_ of 4.5 with Zymo His-Binding buffer mixture and subsequently homogenized. Lysates were clarified by centrifugation. The supernatant was pipetted to a separate tube, and insoluble pellets were resuspended in the same volume of buffer as before lysis. 0.7 μL was taken from either the insoluble pellet resuspension or the supernatant to load on 12% SDS-PAGE (BIO-RAD) for the solubility test (Fig. 3A).

### Activity assays with other noncanonical cofactors

Stock solutions of MNAH (Cayman Chemical) and BNAH were dissolved to 30 mM in pure DMSO before use. The final assay mixture contained 50 mM Tris-Cl buffer pH 7.0 and 0.3 mM of cofactors (final DMSO concentration of 1.0%). The variants’ activities (Fig. 4) were measured by the rate of change in light absorption at 360 nm, using extinction coefficient 6646.43 M^−1^ cm^−1^ for MNAH and 7254 M^−1^ cm^−1^ for BNAH^13^, at 37 °C.

### BNAH synthesis

1-Benzylnicotinamide (BNA^+^) was chemically synthesized as described by Guarneri et al.^5^ with the following modifications: the scale was 20 mmol of nicotinamide and 20 mmol of 1-benzylbromide in 15 mL of acetonitrile. No diethyl ether was added to assist precipitation of the product, which afforded the title compound in an 89% isolated yield (5.24 g, 24 mmol). ^1^H NMR (400 MHz, D_2_O): δ 9.35 (s, 1H), 9.07 (ap d, J = 6.2, 1H), 8.89 (ap d, J = 8.2 Hz, 1H), 8.18 (ap dd, J = 8.2, 6.2 Hz, 1H), 7.49 (s, 5H), 5.89 (s, 2H).

1-Benzyl-1,4-dihydronicotinamide (BNAH) was chemically synthesized as described by Guarneri et al.^5^ with the following modifications: the scale was 6 mmol of 1-benzylnicotinamide, 18 mmol of sodium dithionite, and 12 mmol of sodium carbonate, the reaction procedure took place under argon atmosphere, the product was sufficiently pure not to require recrystallization in a methanol-water mixture, and the product was dried over calcium sulfate instead of phosphorous pentoxide, which afforded the title compound in 60% isolated yield (1.41 g, 11 mmol). ^1^H NMR (400 MHz, DMSO-d_6_): δ 7.32–7.19 (m, 5H), 7.10 (s, 1H), 5.70-5.67 (m, 3H), 4.71-4.68 (m, 1H), 4.23 (s, 2H), 3.11 (m, 2H).

### Modeling *Lp* Nox variants

The homology model of *Lp* Nox was constructed with the I-TASSER webserver^53^. The binding poses of the FAD and NADH ligands were set by aligning the homology model with the related *Lb* Nox crystal structure (PDB: 5VN0)^27^, which shares 65% sequence identity, and transferring the coordinates for the ligands. Final refinement for the binding modes was carried out through Rosetta^54^ relaxation using the FastRelax protocol^55^ to produce an energy minimized model with optimized protein-ligand interactions. The model of the MNAH ligand was downloaded from the PubChem database^56^, and docking was performed by superposition of the nicotinamide ring and carboxamide group with the corresponding atoms in NADH to match the crystal binding pose. A similar binding mode is expected to be necessary for catalysis since MNAH is a truncated version of the native cofactor. Modeling preparations and docking of NMNH were done using Rosetta^57^. Models for the variant *Lp* Nox were produced with Rosetta. Each amino acid substitution was made individually with the MutateResidue mover, then Monte Carlo sampling steps were performed where all residues or ligands within 6 Å of any mutation were allowed to sample alternative rotamer states and backbone dihedrals or torsions to optimize intermolecular interactions. Backbone lever-arm effects were avoided by only making changes in isolated, contiguous residue segments at each move. Local regions were minimized with the EnzRepackMinimize mover^58^ or altered by pivoting a segment using the Backrub mover^59^. 1,000 decoys for each mutant were generated, and the sample with the most favorable total Rosetta score^60^ (an aggregate of hydrophobic, electrostatic, and geometric terms) was selected as the representative model. Example source code are available at GitHub.

### Molecular dynamics simulations

MD simulations of the *Lp* Nox variants were completed with PMEMD from the AMBER20^61,62^ package utilizing the ff14sb^63^ force field and 8 Å Particle Mesh Ewald real space cutoff. Ligand partial charges were determined with the Restrained Electrostatic Potential (RESP)^64^ method at the HF/6-31 G* level using Gaussian09, and other parameters were taken from the General Amber Force Field (GAFF)^65^. Protonation states of titratable residues were determined with the H + + webserver^66^. The TLEAP program was utilized to solvate the complexes with TIP3P^67^ water molecules in a truncated octahedron with 10 Å buffer and 150 mM salt concentration with Na^+^/Cl^-^ counter-ions. The *Lp* Nox systems were minimized in two stages, first with 2,500 steps of steepest descent and 2,500 steps of conjugate gradient where all non-hydrogen solute atoms were restrained with a 20 kcal mol^−1^ Å^−2^ force to relieve solvent clash. The second stage minimization to remove solute steric clashes was run with the same cycle settings and restraints removed. Heating from 0 K to 300 K was performed over 0.5 ns with 10 kcal mol^−1^ Å ^−2^ restraints on all non-hydrogen solute atoms under NPT conditions at 1 atm pressure with Langevin thermostat and 1 fs timestep. Solvent density equilibration was run over 5 ns with 5 kcal mol^−1^ Å^−2^ restraints on all solute atoms and an unrestrained 10 ns equilibration using 2 fs timestep to clear remaining structural artifacts followed the heating stage. Production MD trajectories were each carried out for 400 ns with 2 fs timestep, SHAKE restraints on hydrogens, NVT ensemble, Langevin thermostat with collision frequency 1.0 ps^−1^, and periodic boundary conditions. Two independent replicates were run for the three samples: WT *Lp* Nox with NADH, WT *Lp* Nox with MNAH, and LP 3-EP I158 with MNAH.

### Conformational flexibility and free energy structural analysis

Three metrics were utilized to compare the effects of the mutations on *Lp* Nox conformational dynamics: binding pocket loop flexibility as measured by Root Mean Square Deviation (RMSD) of alpha carbon residues located on loops regions, hydride transfer potential through evaluation of the distance between the nicotinamide ring C4 and FAD N5, and free energy landscape decomposition based on dimensionality reduction with Principal Component Analysis (PCA) of the protein alpha carbon dihedrals. The replicate trajectories were aligned to the reference Rosetta models using alpha carbons for all analysis. Binding pocket loops were defined as residues lining the Rossman fold where the native cofactor NADH is bound. The three regions selected include the positions: 153–157, 177–188, and 238–242. Hydride transfer was categorized into active and inactive binding poses with a 4.5 Å distance cutoff. This was selected as during the simulation the cofactors had the ability to fully dissociate from the protein and sampling large distances would disproportionately influence aggregate values. A cofactor distance of 5 Å would have no material difference in biological effect from 10 Å as both would indicate that the cofactor is too far to participate in catalysis. Phi and psi angles for all alpha carbons were parsed to characterize global conformational dynamics. The data was preprocessed through standard scaling to center the means at zero and variances at one, then transformed through PCA. The two-dimensional free energy landscape was plotted based on the first two principal components, and free energy values were calculated from the density of normalized histogram counts with energy minimum shifted to zero. Structural analysis was completed with the scikit-learn^68^, PyEMMA^69^, pytraj^70^, and PyMOL software packages.

### Reporting summary

Further information on research design is available in the Nature Portfolio Reporting Summary linked to this article.

## Supplementary information

Supplementary Information

Reporting Summary

## Data Availability

All data supporting the finding of this work are available within the paper and its Supplementary Information files. A reporting summary for this paper is available as a Supplementary Information file. Information of strains and plasmids used in this study are available in Supplementary Table 2. Accession codes of crystal structures and genes used in this study are available in Supplementary Table 4. Source data are provided as a Source Data file. Source data are provided with this paper.

## Source data

Source Data

## Code availability

The source code for modeling the conformational dynamics of *Lactobacillus pentosus* NADH oxidase (*Lp* NOX) wildtype and variants utilizing noncanonical cofactor biomimetics are available at GitHub [https://github.com/hanli-lab/nox-eng]. The Rosetta docking protocol for the ligand NMN, ligand FAD and *Lp* Nox LP 7 variant are available at GitHub [https://github.com/siegel-lab-ucd/Orthogonal-glycolytic-pathway-enables-directed-evolution-of-noncanonical-cofactor-oxidase]
