# Supplementary material for: Orthogonal glycolytic pathway enables directed evolution of noncanonical cofactor oxidase
Source: Nat Commun. 2022 Nov 26;13:7282. doi: 10.1038/s41467-022-35021-x (PMC9701214; doi:10.1038/s41467-022-35021-x)
Supplement: Supplementary file 1 — Supplementary Information [file 41467_2022_35021_MOESM1_ESM.pdf]

Orthogonal glycolytic pathway enables directed evolution of noncanonical cofactor oxidase

King, Maxel, and Zhang *et al*.

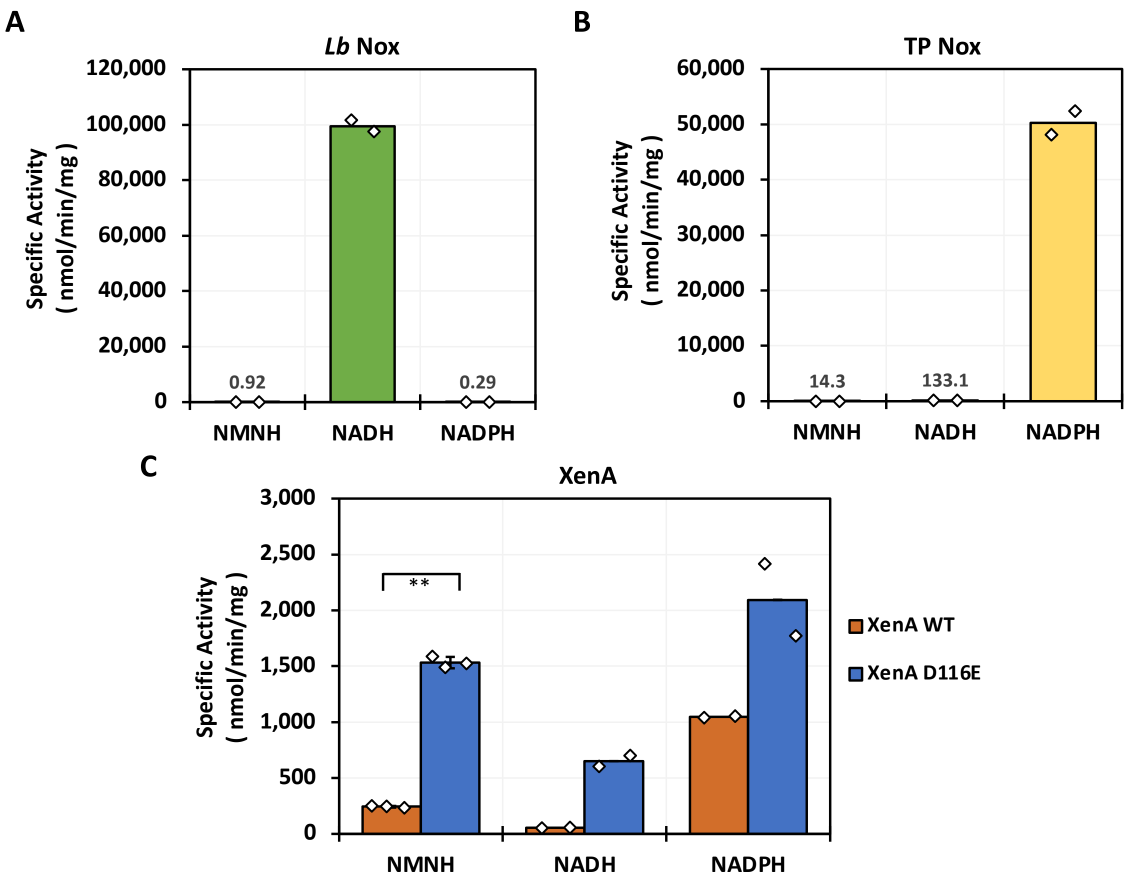

Supplementary Figure 1. Redox partners' specific enzymatic activities towards NMNH, NADH and NADPH at a concentration of 0.3 mM. A, Specific activities of an NADH-specific oxidase from *Lactobacillus brevis* (*Lb* Nox) towards different cofactors. *Lb* Nox (green) only has measurable activity towards its native cofactor, NADH. For all samples, n = 2 biologically independent replicates. **B,** Specific activities of an NADPH-specific oxidase (Triphosphopyridine nucleotide oxidase, TP Nox) towards different cofactors. Similar to *Lb* Nox, TP Nox (yellow) has much higher activity towards its native cofactor, NADPH, than the other two cofactors. For all samples, n = 2 biologically independent replicates. **C,** Broad cofactor specificity of XenA, an enoate reductase from *Pseudomonas putida* XenA WT (orange) has broad cofactor specificity including NMNH compared to *Lb* Nox (green) and TP Nox (yellow). The mutant, XenA D116E (blue), exhibited even higher activity towards NMNH compared to XenA WT (orange). For NMNH-dependent activity assay, n = 3 biologically independent replicates. For all other samples, n = 2 biologically independent replicates. For statistics, XenA WT to XenA D116E, $p = 0.00054$. Data are presented as mean ± standard deviation. Error bars are not displayed for sample size n=2 assay. The statistical significance was determined by two-tail *t*-tests (**: $p < 0.005$). Source data are provided as a Source Data file.

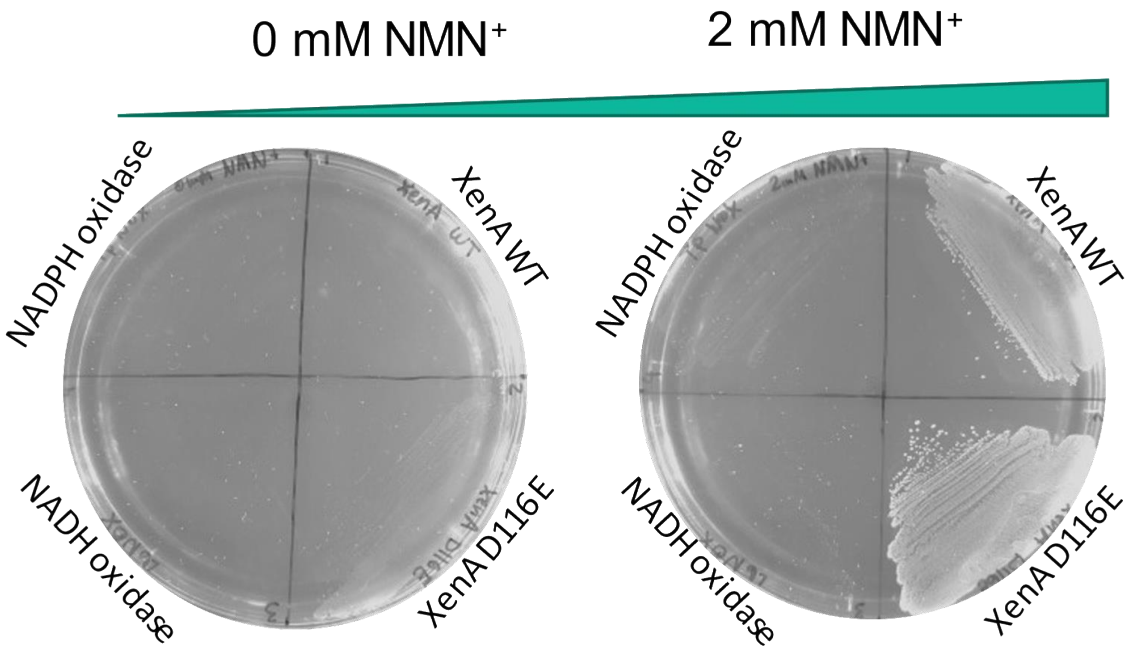

Supplementary Figure 2. Robust growth phenotype of densely plated cells suggests efficient glucose consumption. Selection strain MX502 expressing either *Lb* NOX (NADH oxidase), TP NOX (NADPH oxidase), XenA WT (broad cofactor specificity), or XenA D116E (increased activity compared to WT and broad cofactor specificity) was plated on selection media supplemented with 0, or 2 mM NMN^+ and was incubated at 30° C for an extended growth period (14 days) to heighten contrast of growth between strains expressing NMNH active or inactive redox partners. Supplementation of NMN^+ in media can support minimal conversion of glucose to gluconate. Isolated colonies likely have extensive access to the NMN^+ present to enable growth, while clustered cells are highly limited and required active NMN(H) cycling to support continued growth. However, an active redox partner is essential to efficient and sustained glucose utilization. Although growth rate of single colonies was indicative of NMNH cycling activity, robust clustered growth after re-streaking onto fresh selection media was more predictive of true positives from selection.

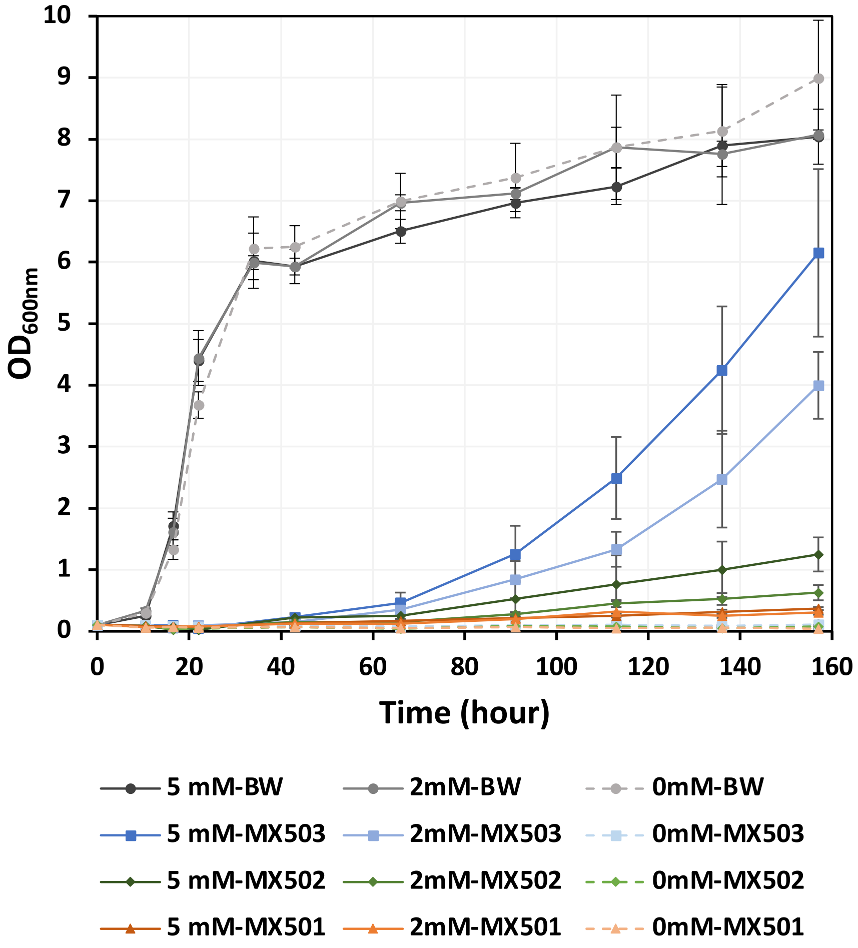

Supplementary Figure 3. Growth rate of selection strains compared to wild-type *E. coli* strain. Growth of wild type (BW25113), MX 501, MX502 and MX503 harboring GDH Ortho and XenA was monitored in M9 minimal media with 20 g/L D-glucose and 0, 2, or 5 mM NMN^+ supplement. All strains possess the gene for initial glucose entry (a glucose facilitated diffusion porin from *Zymomonas mobilis* (*Zm Glf*). Selection strains MX503 (blue square) and MX502 (green diamond) show growth dependence on extracellular NMN^+ supplement while BW 25113 (grey circle) does not. Without heterologous expression of the NMN^+ producing gene (*Francisella tularensis*, *NadEV*) to help built up intracellular NMN^+ pool, MX 501 (orange triangle) is not able to grow, suggesting that *in vivo* NMN^+ biosynthesis still plays an essential role in selection strain growth, even when exogenous NMN^+ is supplied. Notably, while the selection strains exhibited sustained growth and approached similar final cell density to that of wild type, the growth rates were still slow. This may originate from the disruption of the heavily utilized EMP glycolysis, which cannot be fully replaced by the ED pathway, which naturally sustains a much lower carbon flux due to *E. coli*'s regulatory control[1]. For MX501 with 0 or 2 mM NMN^+ supplement, MX503 with 0, 2, or 5 mM NMN^+ supplement, n = 2 biologically independent replicates. For all other samples, n = 3 biologically independent replicates. Data are presented as mean ± standard deviation. Source data are provided as a Source Data file.

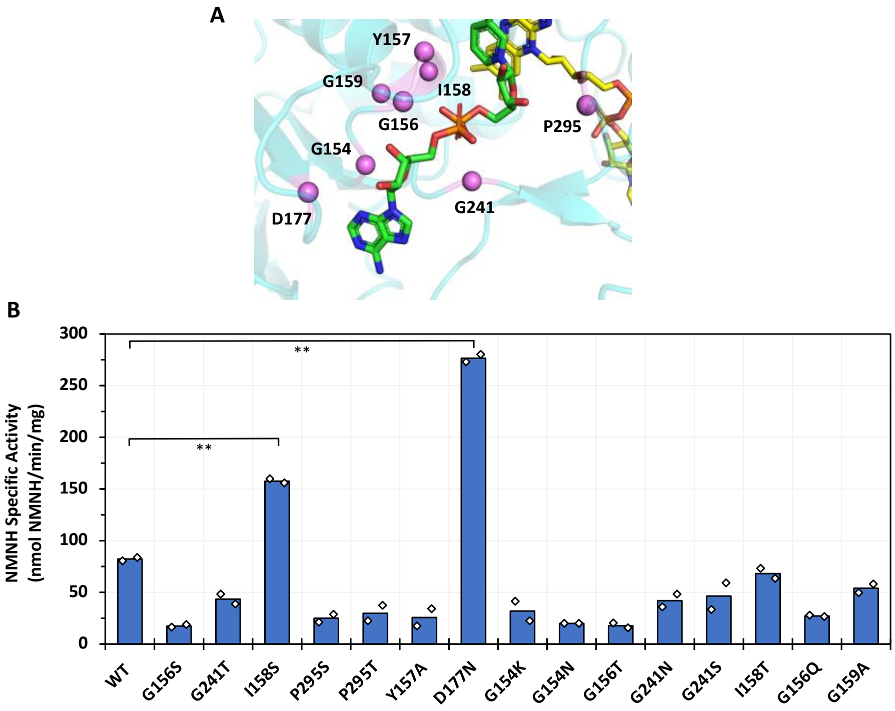

Supplementary Figure 4. Rational design of *Lactobacillus pentosus* NADH oxidase (*Lp* Nox) to utilize NMNH. A, Model of *Lp* NOX positions selected for first round rational design highlighted as purple spheres in *Lp* NOX homology model with NADH (green) and FAD (yellow) bound. D177 is found on the end of the second Rossman beta strand and is known to regulate specificity between NADPH and NADH. Due to its active role discriminating against NADPH, we expect that it may affect NMNH selectivity as well. G154 and G241 are on different loops that compose the adenine cleft, mutations at these positions would alter the volume of the binding pocket and create steric hindrance preventing the larger NADH from fitting, binding of the smaller NMNH is expected to be minimally affected. G156, Y157, I158, and G159 are found at the N-terminal region of the first Rossman alpha helix, these residues align the helix against the NADH pyrophosphate and create a set of polar backbone contacts and positive charge dipole to latch onto the cofactor. Substitutions in this region would reposition the helix to allow more favorable interactions with the NMNH phosphate. P295 is located across the NMNH ribose hydroxyls and mutation may create novel polar interaction. **B,** Specific enzymatic activities of the rational design mutants towards 0.3 mM NMNH. Compared to wild type, I158S and D177N exhibit ~2-fold or ~3-fold increased NMNH activity, respectively. While the mechanism of I158S can be readily rationalized as creating a novel hydrogen bond with the phosphate of NMNH, D177N may be playing a global and indirect role based on its far distance from NMNH. Therefore, D177 is determined as an ideal site for subsequent semi-rational engineering, where I158S is fixed. Compared to the data in Figure 2A, these data were generated using a slightly different method where 0.15mM FAD supplementation in protein purification was omitted. Values represent the mean the two biological replicates. For statistics, WT to I158S, p= 0.0011; WT to D177N, p= 0.00041. The statistical significance was determined by two-tail t-tests (**: $p <$ 0.005). Source data are provided as a Source Data file.

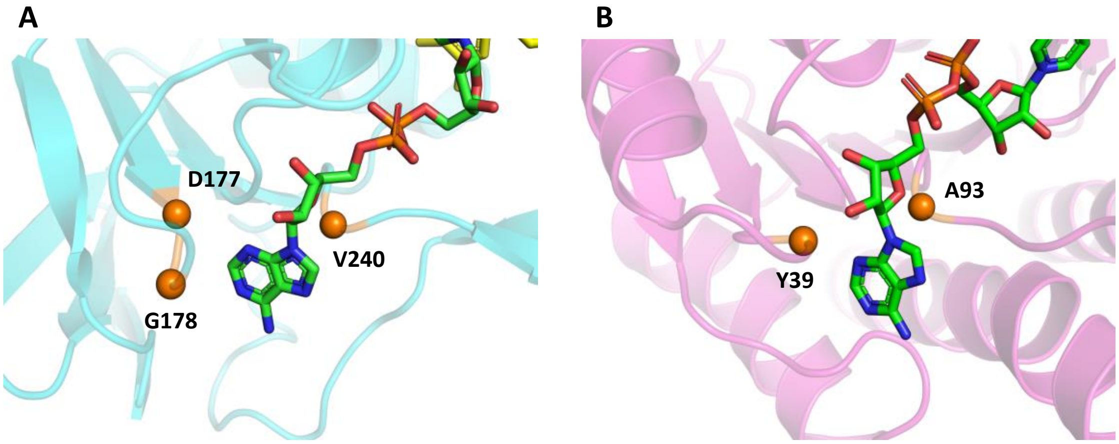

Supplementary Figure 5. Models of *Bs* GDH Triple homology with *Lp* Nox library selected site and Logo plot summarizing mutation frequency at each site. A, Lp NOX library positions highlighted as orange spheres in *Lp* NOX homology model with NADH bound. The positions were selected to be akin to those discovered to enable NMN^+ activity in *Bs* GDH Triple. D177 and G178 are located similarly to Y39 on the specificity loop in GDH, and V240 resembles A93 in packing against the end of the adenosine cleft. **B,** *Bs* GDH Triple homology model with mutated positions that correspond to *Lp* NOX highlighted in orange spheres. Y39 and A93 (orange sphere) enclose the adenosine ring.

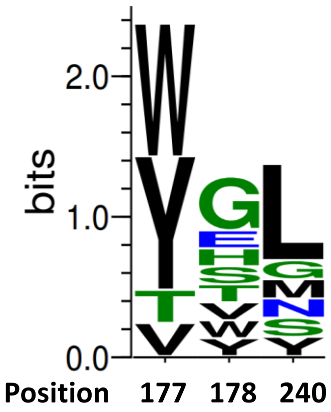

Supplementary Figure 6. Logo plot summarizing the frequency of mutations at each site resulting from library selection. Strong convergence on hydrophobic and bulky residues was observed at positions 177 and 240. Individual variant sequences are available in Supplementary Table 3.

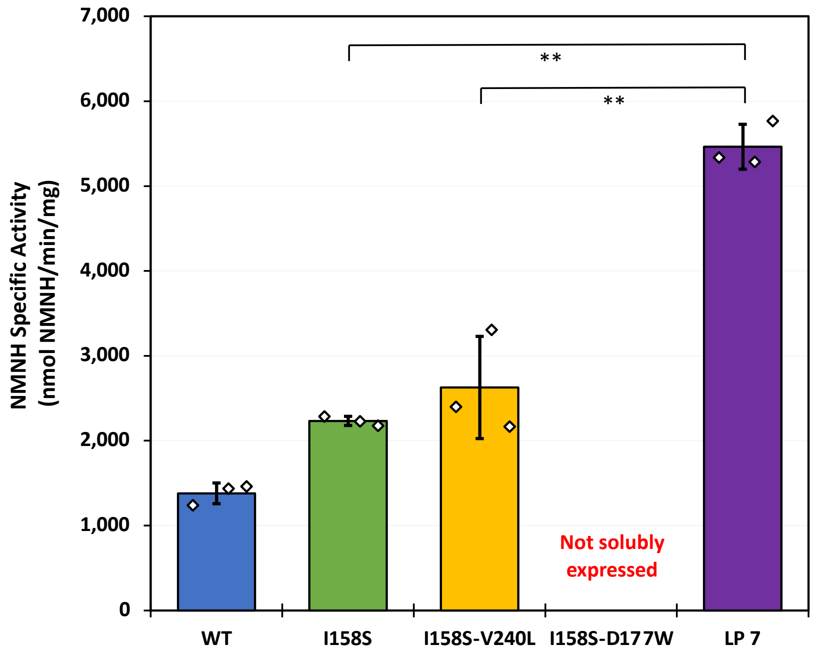

Supplementary Figure 7. The cooperative effect of *Lp* Nox variants' mutations surrounding the adenosine ring. Compared to the wild-type *Lp* Nox (WT), the single mutation I158S increased NMNH-dependent activity. However, adding V240L to I158S did not further increase the activity significantly, and adding D177W to I158S resulted in very poor expression possibly due to impaired protein folding. Importantly, in LP 7 (I158S-D177W-V240L) where both V240L and D177W were combined with I158S, the activity increased ~2-fold compared to I158S, and the expression level was restored. These results are consistent with a non-additive, cooperative effect between V240L and D177W, which are located at the opposing sides of the adenosine binding cleft and predicted to pack against each other via hydrophobic interaction. Our models indicate that the dense packing mitigates *Lp* Nox's excessive flexibility when utilizing the much smaller non-canonical cofactors compared to NADH and promotes robust cofactor binding and catalysis. V240L alone does not afford the activity enhancing effect. The lone hydrophobic mutation D177W on the protein surface may cause a thermodynamic penalty associated with the extra energy needed for its solvation, resulting in poor folding[2,3]. This penalty is compensated by the addition of V240L which forms stabilizing interaction with D177W and makes it more deeply embedded[4,5]. Specific enzymatic activity was measured with NMNH at 0.3 mM. For all samples, n=3 biologically independent replicates. For statistics, I158S to I158S-V240L, p = 0.322, not significant; I158S to LP-7, p = 0.0023; I158S-V240L to LP-7, p = 0.0017. Data are presented as mean $\pm$ standard deviation. The statistical significance was determined by two-tail t-tests (**: $p < 0.005$). Source data are provided as a Source Data file.

Supplementary Table 1. Summary on native and noncanonical cofactors' advantages.

Cofactor	Cofactor class	Advantages	Disadvantages	Cost
**$NAD(P)^+$**	Native	Native biological redox cofactor. Extensive knowledge in engineering for improved activity or redox cofactor specificity switches[6].	Increased cost of biological sourcing[7].	~$1500-$4500/kg[7]
**NMN^+**	Biologically sourced noncanonicals	Truncation of NAD+ offers more structural similarity compared to other noncanonical redox cofactors. This similarity may improve ability to engineer proteins to utilize this cofactor effectively relative to fully synthetic cofactors[8–10]. Established biological synthesis routes enable implementation *in vivo*[9,11,12].	Increased cost of biological sourcing[7].	~$250-$100/kg[7]
**BNA^+**	Synthetic noncanonicals	Facile chemical synthesis methods available leading to reduced input costs[13,14].	High structural deviation requires more extensive engineering relative to NMN^+ [8]. Decreased stability relative to NAD(H)[15].	~$50-$20/kg[7]
**MNA^+**	Synthetic noncanonicals	Facile chemical synthesis methods available leading to reduced input costs[13,14].	High structural deviation requires more extensive engineering relative to NMN^+ [8]. Decreased stability relative to NAD(H)[15].	~$50-$20/kg[7]

Supplementary Table 2. Strains and plasmids used in this study

Strains	Description	Reference
XL-1 Blue	Cloning strain	Stratagene
BL21 (DE3)	Protein expression strain	Invitrogen
BW25113	*E. coli* F-, DE(araD-araB)567, lacZ4787(del)::rrnB-3, LAM-, rph-1, DE(rhaD-rhaB)568, hsdR514	Datsenko *et al.*[16]
DH10β	Electrotransformation strain	Invitrogen
MX103	BW25113 Δ*pncC* Δ*pgi* Δ*zwf* Δ*nadR* Δ*gnd::kan*	Black *et al.*[9]
MX501	BW25113 Δ*pncC* Δ*pgi* Δ*zwf* Δ*nadR* Δ*gnd*	This study
MX502	BW25113 Δ*pncC* Δ*pgi* Δ*zwf* Δ*nadR* Δ*gnd* + pLM106 + pLS502	This study
MX503	BW25113 Δ*pncC* Δ*pgi* Δ*zwf* Δ*nadR* Δ*gnd* ΔpoxB:: kan- P_{LlacO1} *Ft* NadEV	This study
**Plasmids**	**Description**	**Reference**
pCP20	Temperature-inducible yeast Flp recombinase gene controlled by λ*cIts857* in a temperature-sensitive replicon	Datsenko *et al.*[16]
pKD46	*Ori 101 repA101ts* P*BAD gam-bet-exo* Amp^r	Datsenko *et al.*[16]
pQElac	Amp^r ; ColE1 *ori*; P_{LlacO1}. Expression vector	Li *et al.*[17]
pZA	Kan^r ; p15A *ori*; P_{LlacO1}. Expression vector	This study
pRSF	$Spec^r$; RSF *ori*; P_{BAD}. Expression vector	Black *et al.* [9]
pEK102	pQElac 6xHis *Pp* XenA, Amp^r	Black *et al.*[9]
pSM103	pQElac, *Ft* NadEV - *Zm glf* - *Re gntK*, Amp^r	Black *et al.*[9]
pSM105	pQElac *Empty*, Amp^r	Black *et al.*[9]
pLM106	pRSF *Bs* GDH I195R-A93K-Y39Q-S17E, $Spec^r$	Black *et al.*[9]
pLZ204	pQElac *Bs* GDH I195R, Amp^r	Black *et al.*[9]
pLS401	pQElac 6xHis *Lb* Nox, Amp^r	Maxel *et al.*[18]
pLS402	pQElac 6xHis TP Nox (*Lb* Nox G159A-D177A-A178R-M179S-P184R), Amp^r	Maxel *et al.*[18]
pLS501	pQElac 6xHis *Pp* XenA D116E, Amp^r	This study
pLS502	pZAlac, *Ft* NadEV - *Zm glf*, Kan^r	This study
pLS503	pRSF *Bs* GDH I195R-A93K-Y39Q-S17E - *Zm glf*, $Spec^r$	This study
pLS504	pQElac 6xHis *Lp* Nox, Amp^r	This study
pLS505	pQElac 6xHis *Lp* Nox I158S, Amp^r	This study
pLS506	pQElac 6xHis *Lp* Nox I158S-D177X-G178X-V240X, Amp^r	This study
pLS507	pQElac 6xHis *Lp* Nox I158S-D177W-V240L, Amp^r	This study
pLS508	pQElac 6xHis *Lp* Nox I158S-D177W-G178E-V240L, Amp^r	This study
pLS509	pQElac 6xHis *Lp* Nox I158S-D177W-V240L-L179X-Q210X-M211X, Amp^r	This study
pLS510	pQElac 6xHis *Lp* Nox I158S-D177W-V240L-L179H-Q210C-M211W, Amp^r	This study
pLS511	pQElac 6xHis *Lp* Nox I158S-D177W-V240L-L179E-Q210V-M211S, Amp^r	This study
pLS512	pQElac 6xHis *Lp* Nox I158S-D177W-G178E-V240L-P362H-V395L, Amp^r	This study
pLS513	pQElac 6xHis *Lp* Nox D177W-G178E-V240L-P362H-V395L, Amp^r	This study

Abbreviations indicate source of genes: *Bs, Bacillus subtilis; Ft, Francisella tularensis; Lb, Lactobacillus brevis; Lp, Lactobacillus pentosus; Pp, pseudomonas putida; Re, Ralstonia eutropha H16; Zm, Zymomonas mobilis*

Supplementary Table 3. Sequences of *Lp* NOX variants obtained from the first selection.

Variant	Asp 177		Gly 178		Val 240	
	**Codon**	**Residue**	**Codon**	**Residue**	**Codon**	**Residue**
WT	GAC	D	GGT	G	GTC	V
LP 1	TAT	Y	CAT	H	AAT	N
LP 2	TGG	Y	TAT	Y	CTT	L
LP 3	TAT	W	GAG	E	CTG	L
LP 4	TAT	Y	TGG	W	GGG	G
LP 5	TAT	Y	TCG	S	TCT	S
LP 6	GTT	V	ACG	T	ATG	M
LP 7	TGG	W	GGG	G	TTG	L
LP 8	TGG	W	GGT	G	CTG	L
LP 9	TGG	W	GGG	G	CTG	L
LP 10	ACG	T	GTT	V	TAT	Y

Supplementary Table 4. Accession codes of crystal structures and genes used in this study.

Item	Full name	Accession code
Ec Pgi	Glucose-6-phosphate isomerase	Uniprot ID: P0A6T1
Ec Zwf	NADP(+)-dependent glucose-6-phosphate dehydrogenase	Uniprot ID: P0AC53
Ec Gnd	6-phosphogluconate dehydrogenase	Uniprot ID: P00350
Zm Glf	Glucose facilitated diffusion porin	Uniprot ID: P21906
Ec PncC	NMN aminohydrolase	Uniprot ID: P0A6G3
Ec NadR	NMN adenylyltransferase	Uniprot ID: P27278
Ft NadE	NAD(+) synthase	Uniprot ID: Q5NFH5
Ft NadV	Nicotinate phosphoribosyltransferase	Uniprot ID: A0A0E3A662
Bs Gdh	Glucose 1-dehydrogenase	Uniprot ID: P12310
Pp XenA	NADH: flavin oxidoreductase/NADH oxidase	Uniprot ID: Q9R9V9
Lb Nox	FAD-dependent oxidoreductase [Levilactobacillus brevis]	PDB: 5VN0
TP *Nox*	triphosphopyridine nucleotide oxidase	doi: 10.1038/nchembio.2454
Lp Nox	FAD-dependent oxidoreductase	Uniprot ID: F6IXY6

Supplmentary references

1. Hollinshead, W. D. *et al.* Examining *Escherichia coli* glycolytic pathways, catabolite repression, and metabolite channeling using Δpfk mutants. *Biotechnol Biofuels* **9**, 212 (2016).
2. Pakula, A. A. & Sauer, R. T. Reverse hydrophobic effects relieved by amino-acid substitutions at a protein surface. *Nature* **344**, 363–364 (1990).
3. Schindler, T. *et al.* Surface-exposed phenylalanines in the RNP1/RNP2 motif stabilize the cold-shock protein CspB fromBacillus subtilis. *Proteins: Structure, Function, and Genetics* **30**, 401–406 (1998).
4. Frigerio, F., Margarit, I., Nogarotto, R., de Filippis, V. & Grandi, G. Cumulative stabilizing effects of hydrophobic interactions on the surface of the neutral protease from *Bacillus subtilis*. *Protein Engineering, Design and Selection* **9**, 439–445 (1996).
5. Machius, M., Declerck, N., Huber, R. & Wiegand, G. Kinetic Stabilization of *Bacillus licheniformis* α-amylase through introduction of hydrophobic residues at the surface. *Journal of Biological Chemistry* **278**, 11546–11553 (2003).
6. Cahn, J. K. B. *et al.* A general tool for engineering the NAD/NADP cofactor preference of oxidoreductases. *ACS Synth Biol* **6**, 326–333 (2017).
7. Rollin, J. A., Tam, T. K. & Zhang, Y.-H. P. New biotechnology paradigm: cell-free biosystems for biomanufacturing. *Green Chemistry* **15**, 1708 (2013).
8. King, E., Maxel, S. & Li, H. Engineering natural and noncanonical nicotinamide cofactor-dependent enzymes: design principles and technology development. *Curr Opin Biotechnol* **66**, 217–226 (2020).
9. Black, W. B. *et al.* Engineering a nicotinamide mononucleotide redox cofactor system for biocatalysis. *Nat Chem Biol* **16**, 87–94 (2020).
10. Zhang, L. *et al.* Directed evolution of phosphite dehydrogenase to cycle noncanonical redox cofactors via universal growth selection platform. *Nat Commun* **13**, 5021 (2022).
11. Marinescu, G. C., Popescu, R.-G., Stoian, G. & Dinischiotu, A. β-nicotinamide mononucleotide (NMN) production in *Escherichia coli*. *Sci Rep* **8**, 12278 (2018).
12. Black, W. B. *et al.* Metabolic engineering of *Escherichia coli* for optimized biosynthesis of nicotinamide mononucleotide, a noncanonical redox cofactor. *Microb Cell Fact* **19**, 150 (2020).
13. Zachos, I., Nowak, C. & Sieber, V. Biomimetic cofactors and methods for their recycling. *Curr Opin Chem Biol* **49**, 59–66 (2019).
14. Paul, C. E., Arends, I. W. C. E. & Hollmann, F. Is simpler better? Synthetic nicotinamide cofactor analogues for redox chemistry. *ACS Catal* **4**, 788–797 (2014).
15. Nowak, C., Pick, A., Csepei, L.-I. & Sieber, V. Characterization of biomimetic cofactors according to stability, redox potentials, and enzymatic conversion by NADH oxidase from *Lactobacillus pentosus*. *ChemBioChem* **18**, 1944–1949 (2017).
16. Datsenko, K. A. & Wanner, B. L. One-step inactivation of chromosomal genes in Escherichia coli K-12 using PCR products. *Proceedings of the National Academy of Sciences* **97**, 6640–6645 (2000).
17. Li, H. & Liao, J. C. Engineering a cyanobacterium as the catalyst for the photosynthetic conversion of CO2 to 1,2-propanediol. *Microb Cell Fact* **12**, 4 (2013).
18. Maxel, S. *et al.* A growth-based, high-throughput selection platform enables remodeling of 4-hydroxybenzoate hydroxylase active site. *ACS Catal* **10**, 6969–6974 (2020).
