# Supplementary material for: Orthogonal glycolytic pathway enables directed evolution of noncanonical cofactor oxidase
Source: Nat Commun. 2022 Nov 26;13:7282. doi: 10.1038/s41467-022-35021-x (PMC9701214; doi:10.1038/s41467-022-35021-x)
Supplement: Supplementary file 2 — Reporting Summary [file 41467_2022_35021_MOESM2_ESM.pdf]

nature portfolio

Corresponding author(s): Han Li

Last updated by author(s): Nov 11, 2022

Reporting Summary

Nature Portfolio wishes to improve the reproducibility of the work that we publish. This form provides structure for consistency and transparency in reporting. For further information on Nature Portfolio policies, see our Editorial Policies and the Editorial Policy Checklist.

Statistics

For all statistical analyses, confirm that the following items are present in the figure legend, table legend, main text, or Methods section.

n/a	Confirmed	
☐	☒	The exact sample size (*n*) for each experimental group/condition, given as a discrete number and unit of measurement
☐	☒	A statement on whether measurements were taken from distinct samples or whether the same sample was measured repeatedly
☐	☒	The statistical test(s) used AND whether they are one- or two-sided<br>*Only common tests should be described solely by name; describe more complex techniques in the Methods section.*
☒	☐	A description of all covariates tested
☐	☒	A description of any assumptions or corrections, such as tests of normality and adjustment for multiple comparisons
☐	☒	A full description of the statistical parameters including central tendency (e.g. means) or other basic estimates (e.g. regression coefficient) AND variation (e.g. standard deviation) or associated estimates of uncertainty (e.g. confidence intervals)
☐	☒	For null hypothesis testing, the test statistic (e.g. *F*, *t*, *r*) with confidence intervals, effect sizes, degrees of freedom and *P* value noted<br>*Give P values as exact values whenever suitable.*
☒	☐	For Bayesian analysis, information on the choice of priors and Markov chain Monte Carlo settings
☒	☐	For hierarchical and complex designs, identification of the appropriate level for tests and full reporting of outcomes
☒	☐	Estimates of effect sizes (e.g. Cohen's *d*, Pearson's *r*), indicating how they were calculated

Our web collection on statistics for biologists contains articles on many of the points above.

Software and code

Policy information about availability of computer code

Data collection	The starting homology model for Lp NOX was generated with the I-TASSER webserver 5.1.<br>Mutant Lp NOX were created with PyRosetta 2018.52+release.674a9609eaf.<br>The enzymatic assay data were collected with SpectraMax M3 microplate reader.<br>Protein structures were visualized with Schrodinger PyMOL 2.3.4.<br>Molecular dynamics simulations were performed with AMBER 2020 and analyzed with PyEMMA 2.5.7 and pytraj 2.0.4.
Data analysis	General data analysis was performed with python 3.7 and the packages: numpy 1.19.1, matplotlib 3.2.1, pandas 0.24.2, scikit-learn 0.23.2 and Microsoft Excel 16 for Windows and Microsoft Excel 16.58 for Mac.

For manuscripts utilizing custom algorithms or software that are central to the research but not yet described in published literature, software must be made available to editors and reviewers. We strongly encourage code deposition in a community repository (e.g. GitHub). See the Nature Portfolio guidelines for submitting code & software for further information.

Data

Policy information about availability of data

All manuscripts must include a data availability statement. This statement should provide the following information, where applicable:

- Accession codes, unique identifiers, or web links for publicly available datasets
- A description of any restrictions on data availability
- For clinical datasets or third party data, please ensure that the statement adheres to our policy

There is no restriction on data availability. All data supporting the finding of this work are available within the paper and its Supplementary Information file. Source data are provided with this paper. The table of strains and plasmid used in this study has been deposited in the Supplementary Information. Supplementary data 1

provides accession codes of crystal structures and genes used in this study. The source code for modeling the conformational dynamics of Lactobacillus pentosus NADH oxidase (Lp NOX) wildtype and variants utilizing noncanonical cofactor biomimetics are available at GitHub [https://github.com/hanli-lab/nox-eng]. The Rosetta docking protocol for the ligand NMN, ligand FAD, and Lp Nox LP 7 variant is available at GitHub [https://github.com/siegel-lab-ucd/Orthogonal-glycolytic-pathway-enables-directed-evolution-of-noncanonical-cofactor-oxidase].

Field-specific reporting

Please select the one below that is the best fit for your research. If you are not sure, read the appropriate sections before making your selection.

☒ Life sciences ☐ Behavioural & social sciences ☐ Ecological, evolutionary & environmental sciences

For a reference copy of the document with all sections, see nature.com/documents/nr-reporting-summary-flat.pdf

Life sciences study design

All studies must disclose on these points even when the disclosure is negative.

Sample size	All experiments were performed at least in triplicate except for the initial screening process for the protein variants, where sample size n=2. Sample size choice is consistent with previous studies in this field (Main text ref -14,26). No specific calculation or statistical analysis was performed to determine the sample size for each experiment.
Data exclusions	No data were excluded.
Replication	All replication attempts were successful. Every experiment with unknown candidates was performed with controls with known activity as chaperons, and controls were consistent with previously known results. All experiments are independent.
Randomization	All cultures and samples in the same experiment were treated identically. Therefore, randomization was not relevant.
Blinding	This study reports objective measurements of samples derived from bacterial culture, purified proteins, or chemical reactions. In each experiment, the samples were treated identically. Therefore, blinding was not relevant.

Reporting for specific materials, systems and methods

We require information from authors about some types of materials, experimental systems and methods used in many studies. Here, indicate whether each material, system or method listed is relevant to your study. If you are not sure if a list item applies to your research, read the appropriate section before selecting a response.

Materials & experimental systems

n/a	Involved in the study
☒	☐ Antibodies
☒	☐ Eukaryotic cell lines
☒	☐ Palaeontology and archaeology
☒	☐ Animals and other organisms
☒	☐ Human research participants
☒	☐ Clinical data
☒	☐ Dual use research of concern

Methods

n/a	Involved in the study
☒	☐ ChIP-seq
☒	☐ Flow cytometry
☒	☐ MRI-based neuroimaging
